# Synthesis, Physicochemical Properties, and Ion Recognition Ability of Azulene-Based Bis-(Thio)Semicarbazone

**DOI:** 10.3390/molecules30010083

**Published:** 2024-12-29

**Authors:** Anamaria Hanganu, Catalin Maxim, Andreea Dogaru, Adrian E. Ion, Coralia Bleotu, Augustin M. Madalan, Daniela Bala, Simona Nica

**Affiliations:** 1 “C. D. Nenitzescu” Institute of Organic and Supramolecular Chemistry, Splaiul Independentei 202B, 060023 Bucharest, Romania; anamaria_hanganu@yahoo.com (A.H.); catalin.maxim@chimie.unibuc.ro (C.M.); g.a.dogaru@gmail.com (A.D.); eugeniu_ion@yahoo.com (A.E.I.); 2 Faculty of Chemistry, University of Bucharest, 4-12 Bvd. Regina Elisabeta, 030018 Bucharest, Romania; augustin.madalan@chimie.unibuc.ro (A.M.M.); dbala@gw-chimie.math.unibuc.ro (D.B.); 3 Stefan S. Nicolau Institute of Virology, 285 Mihai Bravu Avenue, 030317 Bucharest, Romania; cbleotu@yahoo.com; 4 Department of Botany and Microbiology, Faculty of Biology, University of Bucharest, 1-3 Aleea Portocalelor, 060101 Bucharest, Romania

**Keywords:** azulene-bis(thio)semicarbazone, single-crystal X-ray crystallography, spectroscopic characterization, redox properties, cytotoxicity

## Abstract

Azulene-1,3-bis(semicarbazone), **1**, and azulene-1,3-bis(thiosemicarbazone), **2**, were synthesized by the acid-catalyzed condensation reactions of semicarbazide and thiosemicarbazide, respectively, with azulene-1,3-dicarboxaldehyde in stoichiometric amounts. Compounds **1** and **2** were identified by high-resolution mass spectrometry and characterized by IR, ^1^H-NMR, ^13^C-NMR, and UV-vis spectroscopic techniques. Crystal structure determination of azulene-1,3-bis(thiosemicarbazone) shows that the thiosemicarbazone units exhibit a *syn*-closed conformation, with both arms oriented in the same direction and adopting an *E* configuration with respect to the imine linkages. Both hydrazones are redox active and showed fluorescence emission at 450 nm upon excitation at 350 nm. The bis-semicarbazone showed no affinity for anions nor for mercury(II) metal cation. Instead, the bis-thiosemicarbazone showed a lower affinity for chloride anions, but enhanced affinity for binding/poisoning Hg^2+^ ions. Both compounds were tested against osteosarcoma MG63 cell lines, exhibiting low antiproliferative activity with comparable IC_50_ values of 473.08 μM and 472.40 μM for compounds **1** and **2**, respectively. Despite this limited antiproliferative effect, further analysis using propidium iodide staining revealed a concentration-dependent decrease in cell viability, with high concentrations inducing a marked reduction in cell number, accompanied by morphological changes characteristic of apoptosis and necrosis.

## 1. Introduction

Azulene derivatives have garnered significant interest due to their applications in various fields, including medicinal chemistry [1], materials science [2,3], photoswitches [4,5], and organic electronics [6,7], where their distinct structural and electronic features enable novel functionalities and reactivity patterns [8]. Azulene is a blue-colored hydrocarbon with unusual electronic behavior such as a dipole moment [9] and visible light absorption owing to its structural fused five- and seven-membered ring system [10]. Azulene exhibits interesting redox properties, being able to undergo electrochemical oxidation and reduction [11,12], in most cases as reversible processes with promising potential in electrochemical sensing and cytotoxicity for cancer treatment [13]. Distinct from many other aromatic hydrocarbons, azulene exhibits fascinating fluorescence observed from the second excited singlet state (S_2_), which is an exception to Kasha’s rule [14]. The fluorescence properties can be significantly altered by substituting different functional groups onto the azulene core, azulene derivatives being promising for developing fluorescence-based sensors [15]. Furthermore, azulene derivatives have shown a range of biological activities ranging from anti-inflammatory to anti-cancer properties which are explored in drug development and medical treatments [16,17,18,19,20].

The aromatic core of azulene allows for strong π–π interactions, while the functional groups attached to it, such as amino, carboxyl, or hydroxyl groups, can be tailored to interact with specific anions or metal ions [21,22]. This ability to finely tune the recognition sites makes azulene derivatives ideal candidates for the development of chemosensors for environmental monitoring, especially in detecting harmful substances like heavy metal ions [23]. Azulene derivatives have been successfully employed as chemosensors for detecting toxic anions such as fluoride (F^−^), cyanide (CN^−^), and other hazardous species [24,25]. The conjugated azulene system, combined with electron-donating or electron-withdrawing substituents, allows for significant changes in their optical properties upon interaction with anions. These changes are often easily detectable via UV-Vis or fluorescence spectroscopy, making azulene-based sensors practical tools for real-time environmental monitoring [15].

We thought it worth designing novel azulene-based semicarbazone and thiosemicarbazone with the aim of interplay between the azulene scaffold and the chelating ability of the semicarbazone or thiosemicarbazone groups. Hydrazone has emerged as a significant building block in coordination chemistry [26,27] or found applications in catalysis [28,29] and medicinal chemistry [30,31,32]. Particularly, thiosemicarbazones have been widely studied for their potential biological activities, including antibacterial [33,34], antifungal [35], and anticancer properties [36]. Thiosemicarbazones have also been used as chelating agents [37,38], catalysts [39], and sensors [40]. However, while significant progress has been made in the study of mono-(thio)semicarbazone-based compounds, the exploration of bis-(thio)semicarbazones remains a relatively underdeveloped area of research. These types of compounds are versatile building blocks for construction of metal complexes with a large variety of properties ranging from molecular magnets [41] to catalysts [42] or biological active compounds [43]. Bis-thiosemicarbazones have been investigated as anion receptors, with some exhibiting the ability to detect specific anions with the naked eye [44]. The anion binding supposes hydrogen bonding interactions between the thiourea moiety and the guest anions. In addition, the presence of donor atoms such as nitrogen and sulfur (in thiosemicarbazones) or nitrogen and oxygen (in semicarbazones) enables these compounds to form stable coordination complexes with toxic heavy metals, including mercury (Hg^2+^), cadmium (Cd^2+^), and lead (Pb^2+^) [44].

Inspired by the distinctive visible absorption band of azulene derivatives, we designed and synthesized azulene-bis(semicarbazone), **1**, and thiosemicarbazone, **2,** compounds, and evaluated their potential as receptors for anions and toxic mercury(II) cation, using UV-Vis and ^1^H-NMR spectroscopy. Additionally, we took advantage of the unique structural features of azulene to explore the fluorescent and electrochemical properties of these compounds, and also assessed their potential anticancer activities on osteosarcoma cell lines.

## 2. Results and Discussion

### 2.1. Synthesis and NMR Characterization

**Scheme 1:**
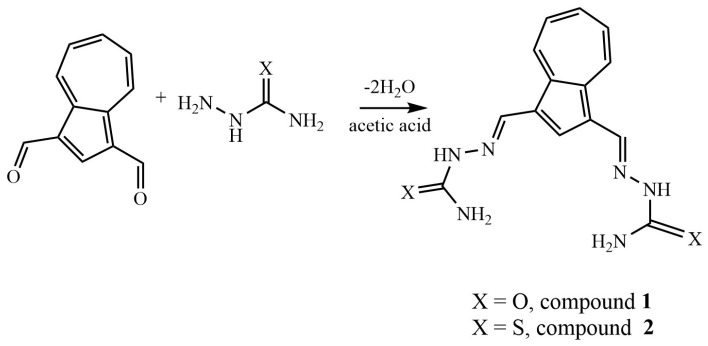
Synthesis and schematic representation of the azulene-1,3-bis(hydrazones).

The azulene-bis(hydrazone) **1** and **2** were synthesized via straightforward Schiff base condensation reaction between azulen-1,3-dicarboxaldehyde and commercially available semicarbazide and thiosemicarbazide, respectively (Scheme 1). The reaction proceeds in the presence of glacial acetic acid as a catalyst, utilizing an excess of semi- and thiosemicarbazide (2.5 equivalents), in refluxing ethanol. The identification of the products was carried out by high-resolution mass spectrometry.

**Figure 1:**
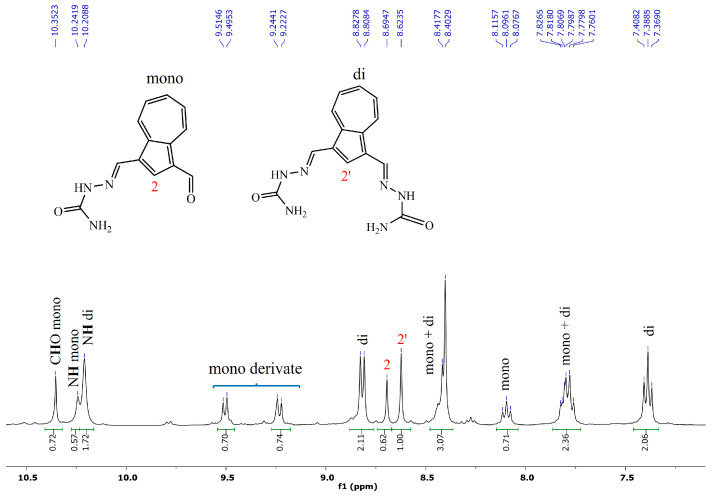
^1^H-NMR spectra of the reaction mixture obtained by Schiff base condensation reaction in the absence of acetic acid.

The reaction protocol was established for the Schiff base condensation of azulene-1,3-dicarboxaldehyde and semicarbazide. In the absence of acetic acid, a mixture of mono- and di-substituted semicarbazone was obtained in a ratio of 0.5:1, as confirmed by NMR spectroscopy. The ^1^H NMR spectroscopy showed the aldehyde proton (CH=O) at 10.35 ppm and two distinct signals corresponding to the imine proton (CH=N) at 8.40 and 8.38 ppm (Figure 1). Integration of the proton signals enabled determination of the molar ratio of the two reaction products present in the mixture.

**Figure 2:**
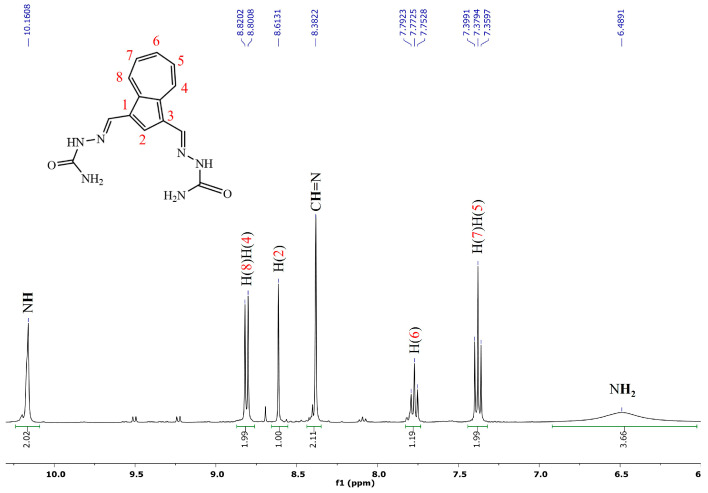
^1^H-NMR spectra of azulene-1,3-bis(semicarbazone), **1**, in DMSO-d_6_ (500 MHz).

In the presence of a few drops (five) of acetic acid, the reaction preferentially yields the desired symmetric compound. The reaction time was determined based on the disappearance of azulene-1,3-dicarboxaldehyde from the reaction medium via thin-layer chromatography. The ^1^H NMR spectrum of compound **1** shows the chemical shifts characteristic of the azulene moiety within the range of 7.36–8.82 ppm area. The N*H* protons are observed at 10.17 ppm, as a well-defined signal, while the two N*H*_2_ protons resonated at 6.49 ppm as a broad singlet. The characteristic C*H*=N proton resonance appears at 8.38 ppm in the shape of a well-defined singlet (Figure 2). The ^13^C NMR spectrum shows the characteristic azulene carbon resonance signals, the amide carbonyl peak at 156.9 ppm, and imine carbon resonance at 135.6 ppm.

**Scheme 2:**
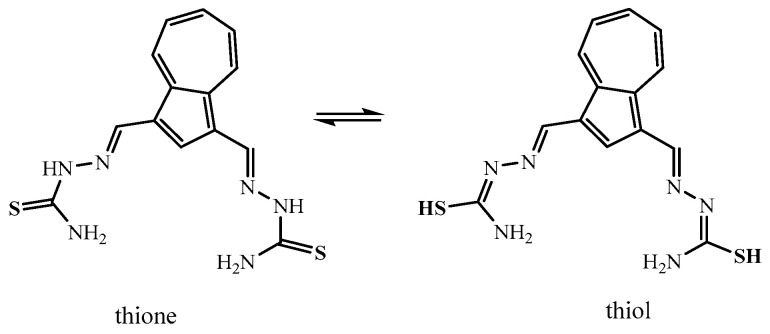
Schematic representation of possible thione–thiol tautomerism in azulene-1,3-bis(thiosemicarbazone), **2**.

**Figure 3:**
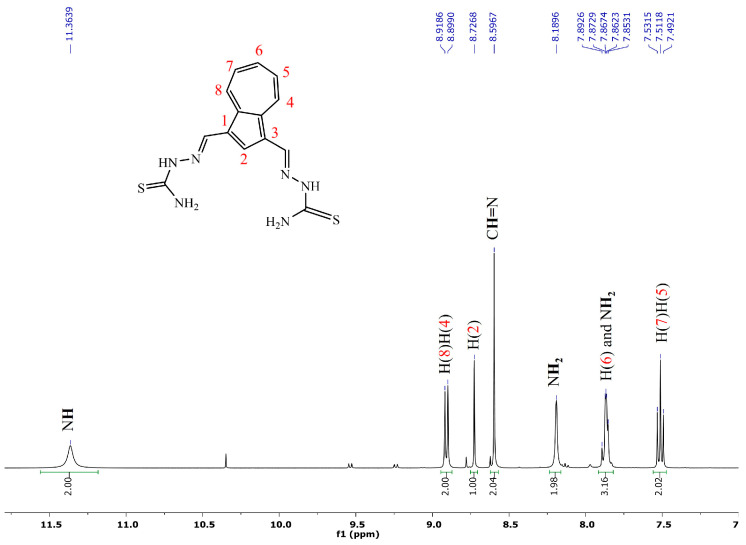
^1^H-NMR spectrum of azulene-1,3-bis(thiosemicarbazone), **2**, in DMSO-d_6_ (500 MHz).

For the thiosemicarbazone compound **2**, the imine proton exhibits a downfield shift to 8.60 ppm, whereas the N*H*_2_ protons appear as two distinct, broad singlets at 8.20 and 7.86 ppm, respectively (Figure 3), due to electron delocalization and the possible thione–thiol equilibrium in solution (Scheme 2). The reduced electronegativity of the sulfur likely contributes to this phenomenon. The N*H* protons also exhibit a significant shift, with its resonance occurring at 11.23 ppm, which represents a downfield shift of over 1 ppm from its position in compound **1**. However, in the ^13^C-NMR spectrum of the compound, the corresponding signal appears at 177.13 ppm, corresponding to the thioamide carbon (C=S) (Figure S2, Supplementary Materials). Thus, the ^13^C-NMR spectrum and the presence of the N*H* resonance collectively indicate that the thioenol form is not present. The two distinct singlets corresponding to C(=S)–N*H*_2_ protons suggest that the protons are magnetically nonequivalent, likely due to hindered rotation around the C-N bond.

### 2.2. Single-Crystal X-Ray Structure Determination and IR Spectroscopy

**Figure 4:**
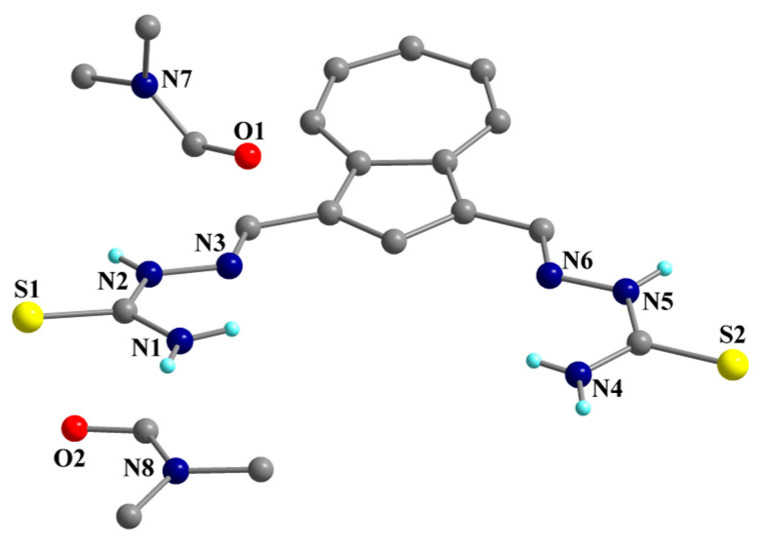
Asymmetric unit of compound **2**: one molecule of the thiosemicrabazone and two molecules of DMF; selected atom labeling is given; non-relevant H-atoms are omitted for clarity.

The structure of compound **2** was confirmed definitively by single-crystal X-ray analysis. X-ray diffraction was performed at room temperature on a single crystal grown from a solution of this compound in a solvent mixture of methanol/dimethylformamide (MeOH/DMF) by slow solvent evaporation. Crystal data, details for data collection, and parameters for structure solution and refinement are summarized in Table 1. This compound crystallized in the monoclinic *P*2_1_/*c* space group with *Z* = 4. The crystallographic asymmetric unit comprises one thiosemicarbazone molecule together with two DMF molecules of crystallization as depicted in Figure 4. Selected bond distances are presented in Table 2. The isolation of thiosemicarbazone as the thione tautomer is demonstrated by the thiocarbonyl (C=S) bond distances [C1–S1 = 1.691(3) Å and C14–S2 = 1.688(3) Å], which are characteristic of thioamide double bonds [45]. Interestingly, the Schiff-base imine bonds C2–N3 and C13–N6 are identical [1.284(4) Å].

The N-N bonds are 1.368 and 1.3916 A, similar to other thiosemicarbazones and longer than the N-N bonds in hydrazide (1.325 Å). The thiosemicarbazone units exhibit a *syn*-closed conformation, with both arms oriented in the same direction and adopting an *E* configuration with respect to the imine linkages. The conformation of the arms is in contrast to the bis-thiosemicarbazone derived from 2-hydroxyisophthalaldehyde, where intramolecular hydrogen bonding between the imine nitrogen and phenolic oxygen leads to an *anti* arrangement of the arms [46].

**Figure 5:**
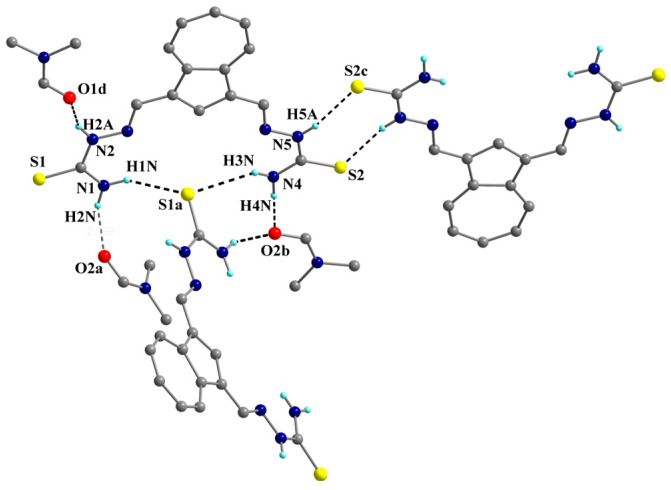
Intermolecular hydrogen bonds in crystal structure of **2**; symmetry code: ^a^ = x, 1/2 − y, −1/2 + z; ^b^ = x, y, −1 + z; ^c^ = 1 − x, 1 − y, −z; ^d^ = x, 1/2 − y, −1/2 + z.

**Figure 6:**
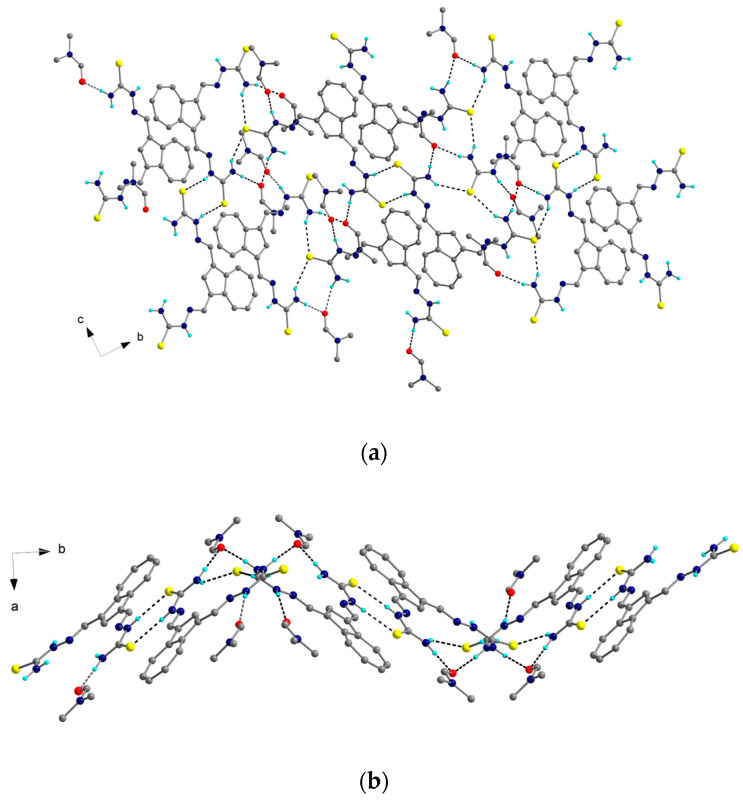
2D sheets in crystal structure of **2** as viewed along crystallographic *a* axis (**a**) and along crystallographic *c* axis (**b**).

**Figure 7:**
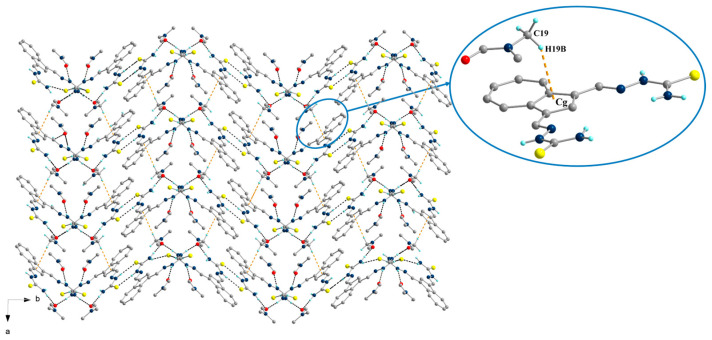
Crystal packing diagram of compound **2** as viewed along a axis; inset: detail of the C-H···π interactions.

Intermolecular N-H···O and NH···S hydrogen bonds were observed in the crystal packing (Figure 5). Additionally, the intermolecular NH···S contacts form the R^2^_2_(8) closed ring according to the symmetry center, {···H–N–C=S}_2_ synthons. The molecules are bonded to each other with these interactions into 2D sheets (Figure 6), which are further connected via C-H···π interactions (Figure 7). The data related to the hydrogen bonds are given in Table 2.

The IR spectroscopy of both compounds revealed characteristic bands corresponding to v(N–H), ν(C=N), ν(N–N), ν(C=O), and ν(C=S) stretching vibrations (see Section 3 and Figures S3 and S4 in the Supplementary Materials). Notably, the imine stretching vibration was observed at 1577 cm^−1^ for compound **1** and 1591 cm^−1^ for compound **2**. The C=O stretching vibration appeared as a sharp, intense band at 1676 cm^−1^, while the C=S stretching vibration was observed at 831 cm^−1^. Furthermore, the absence of the 2500 cm^−1^ band, characteristic of v(S-H), in the IR spectrum of compound **2** confirmed the thione form (HN–C=S) in the solid state, as supported by X-ray crystallography. On the other hand, the presence of a ν(N–H) stretching vibration at 3121 cm^−1^ provides evidence for the azo–hydrazone tautomer of the hydrazone group.

### 2.3. Electronic Absorption Spectroscopy and Fluorescence

UV-Vis spectra for both compounds display absorption maxima at 331 nm (lgɛ = 4.79) for compound **1** and 360 nm (lgɛ = 4.90) for compound **2**. A less intense absorption peak is present in the UV region for compound **1**, at 296 nm. Both compounds showed a broad absorption peak at 410 nm (Figure S5, Supplementary Materials). In a solid state, broad peaks are observed at 450 and 600 nm, characteristic of the azulene-visible absorption peak near 600 nm, which is responsible for its blue color [15] (Figure S6, Supplementary Materials).

**Figure 8:**
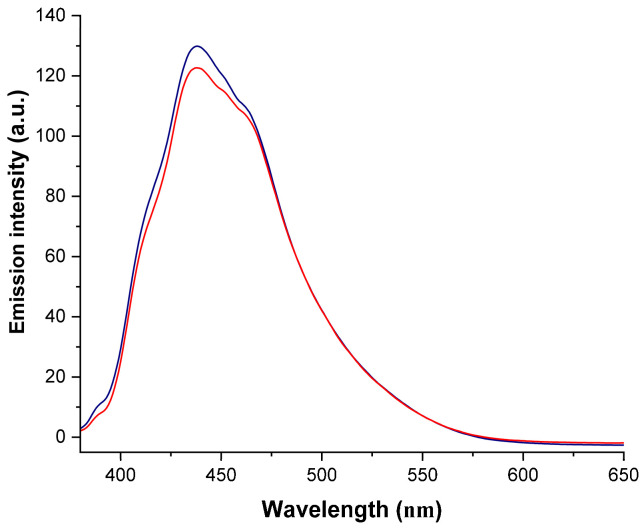
Emission spectra of compounds **1** (3.48 × 10^−6^ M, blue) and **2** (3.34 × 10^−6^ M, red) in DMSO solution at room temperature.

Both compounds showed fluorescent emission upon excitation at a 350 nm wavelength (Figure 8). The fluorescence emission occurs around 450 nm for both compounds, similar to the emission spectra of the parent azulene when it relaxes from the S_2_ state to the ground state [14]. It is well known that the S_2_→S_0_ fluorescence of azulenes is much stronger compared to the weak S_1_→S_0_ transition and it is detectable in the visible region (blue fluorescence) [14]. The relative fluorescence quantum yield is 0.11 for **1** and 0.07 for **2**, referred to as standard quinine sulphate fluorescence in acidic medium.

### 2.4. Electrochemistry

Azulene derivatives often exhibit interesting redox properties, making them potential candidates for applications in electrochemistry and materials science [47].

**Figure 9:**
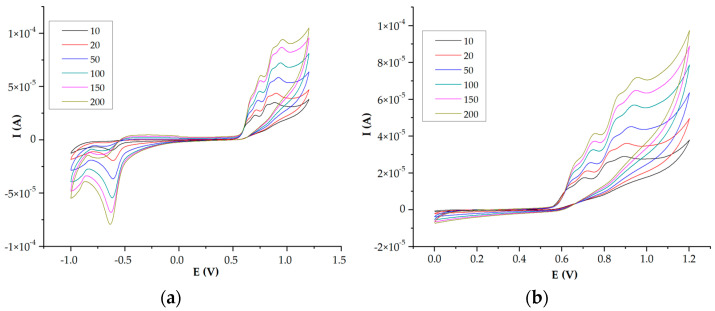
The cyclic voltammograms of 1 mM compound **1** over the potential range [0–1.2–(−1)] (**a**) and [0–1.2–0] (**b**) at scan rates 10–200 mV/s.

The redox properties of the azulene-derived semicarbazone, **1**, were investigated by cyclic voltammetry (CV) over a broad potential range of [0–1.2–(−1)] V (Figure 9a) and over a narrower potential range, [0–1.2–0] V, in order to facilitate the correlation of anodic and cathodic peaks (Figure 9b).

**Figure 10:**
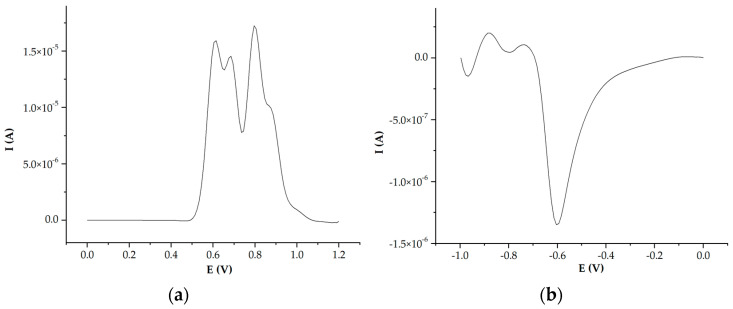
DPV traces of 1 mM compound **1** in DMSO, with SP = 10 mV and MA = 25 mV in the 0.0 V to 1.2 V (**a**), and 0.0 to −1.0 V (**b**) potential range, scan rate 20 mV/s.

Within the potential range from 0 to −1 V and back to 0, no oxidation peaks were observed (see Supporting Information Figure S7). In contrast, oxidation peaks are detected in the other two studied domains at 0.71 and 0.82 V. Two reduction peaks are observed at approximately −0.8 V (visible in DPV measurements), which are attributed to the reduction of the imine double bond, and at −0.60 V, ascribed to the reduction of the azulene moiety. The cyclic voltammetry data are well correlated with the DPV measurements (Figure 10).

The assignment of the oxidation and reduction peaks, both the cyclic voltammetry and DPV experiments, were performed for related phenylene-derived hydrazones, measured in the [0–1–(−0.85)] range (see Supporting Information Figure S8). The benzene ring is significantly more stable and therefore the redox behavior is as well due to the semicarbazone moiety. The anodic peak is very poorly defined (not visible on CV measurements), while the cathodic peaks in the potential range of −0.3 to −0.85 V are not present, indicating that reduction occurs with greater difficulty. Nevertheless, a comparison of the redox potentials of the two compounds enables the assignment of the oxidation and reduction potentials of azulene, as described above.

**Figure 11:**
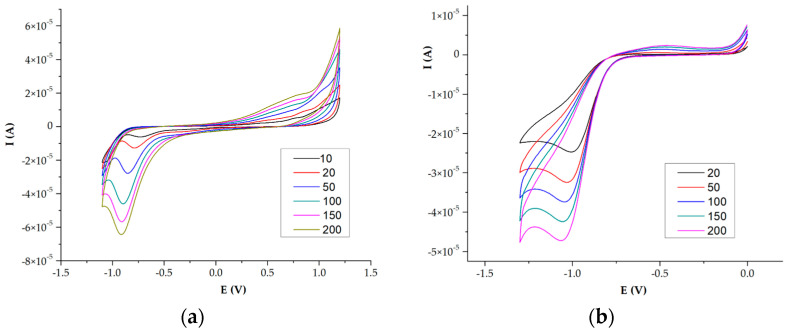
The cyclic voltammograms of 1 mM compound **2** over the potential range: (**a**) [0–1.2–(−1.1)] and (**b**) [0–(−1.3)–0] at scan rates 10–200 mV/s.

**Figure 12:**
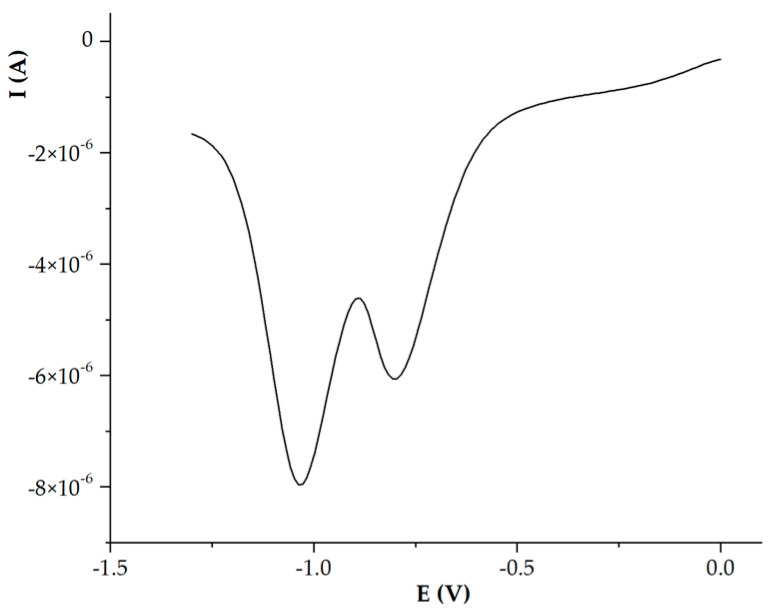
DPV trace of 1 mM compound **2** in DMSO, with SP = 10 mV and MA = 25 mV in the 0–(−1.3) V potential range, scan rate 20 mV/s.

The substitution of oxygen with a sulfur atom, as in thiosemicarbazone **2**, yields a more stable compound. No oxidation process was observed, and the reduction occurs at around −0.8 V, which is slightly more difficult to achieve, likely due to the increased electronegativity of sulfur (Figure 11a,b). The reduction peak potential is shifted to a more negative value with increasing scan rate. From DPV measurements, two cathodic peaks are observed at −0.75 V and −1 V (Figure 12).

The reduction peak potentials for compound **2** are shifted to more negative values compared to those of compound **1**, indicating higher stability of thiosemicarbazone. Similar measurements were conducted for phenylene–thiosemicarbazone in the potential range of [0–1–(−1)] (see Supporting Information, Figure S9). When azulene was substituted with benzene, no oxidation process was observed, and a small reduction peak appeared at 0.9 V.

### 2.5. Receptor Ability

Next, we tested the ability of these compounds to function as a receptor for anions, owing to the good hydrogen bond donor ability of the N-NH-C=O(S)hydrazone and the iminothiourea moiety. Due to the optical properties of the azulene chromophore, we hoped that our systems could signal the presence of specific anion guests by changes in color. It turned out that the interactions could not be detected by simple naked-eye observation of the solutions of these receptors with anions. Next, we carried out the UV-Vis titration experiments to quantitatively determine the receptors’ affinities toward anions. The UV-Vis spectra of the azulene-containing semicarbazone and thiosemicarbazone receptors **1** and **2** displayed prominent absorption bands at 330 nm and 360 nm, respectively, in DMSO as a solvent, characteristic of the π-π* transition of the azulene rings. Solution binding studies were performed by UV-Vis titrations using [n-Bu_4_N]^+^Cl^−^ (Figure S10, Supplementary Materials). For both hydrazones, no relevant spectral changes were observed in the presence of the chloride anion, only a slight decrease in the absorption intensity as a result of dilution upon the addition of the anion solution.

**Figure 13:**
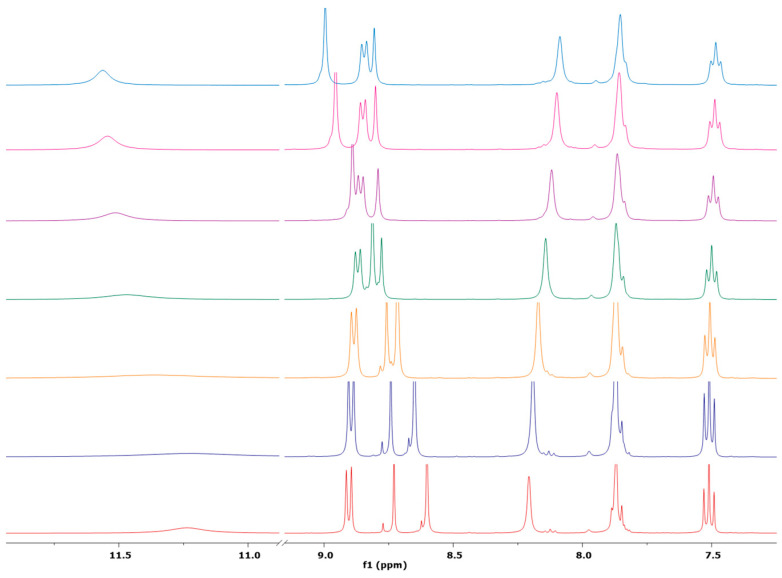
^1^H-NMR spectra of **2** (10 mM) in DMSO-*d*_6_ with solid TBACl (0, 1, 2, 4, 6, 8, and 10 equivalents).

Given the low concentrations employed in the UV-Vis titrations, where hydrogen bonding interactions are significantly weaker, we complemented our studies with NMR spectroscopy, which operates at much higher concentrations, thereby enhancing our ability to detect subtle interactions. The anion binding ability of **1** was very poor and tested only for Cl^−^ and Br^−^ (Figures S11 and S12 in the Supplementary Materials). While the addition of a bromide anion caused no significant changes in the position of the proton resonances, in the case of the chloride anion, a small downfield shift of the NH peaks was observed (ΔδNH = 0.23 ppm), exhibiting a modest recognition effect. Thus, detailed studies were taken using ^1^H-NMR spectroscopy, adding solid [n-Bu_4_N]^+^X^−^ (X^−^ = Cl^−^, Br^−^, ClO_4_^−^, HSO_4_^−^, BF_4_^−^, and PF_6_^−^) to DMSO-*d*_6_ solution of azulene-based thiosemicarbazone, **2**. For this compound, the NH resonance shifts after the addition of a chloride anion from 11.23 in free thiosemicarbazone to 11.56 ppm after the addition of 6 equivalents of solid tetra-butylammonium chloride, with no further significant changes upon increase of the anion concentration to 10 equivalents (Figure 13). The two resonances of the NH_2_ groups (NH_2_1 = 8.20 ppm and NH_2_2 = 7.86 ppm) shift upfield (ΔδNH_2_1 = 0.12 ppm) and the NH_2_2 proton shifts upfield (ΔδNH_2_2 = 0.02 ppm) upon anion coordination. The calculated association constant using the http://app.supramolecular.org/bindfit/ (accessed in October 2024) software [48] was found to be 32.13 M^−1^, indicative of a weak recognition effect for chloride anions.

In the case of the bromide anion, the shifts of the NH and NH_2_ protons are less significant, thus having a lower affinity (Figure S13, Supplementary Materials). Analysis of the crystal structure shows the NH groups outside of the binding cleft, being involved in N-H···O and NH···S hydrogen bonding interactions. Notably, the sulfur and nitrogen donor atoms (N3/S1 and N6/S2) are positioned in an anti-parallel fashion; thus, the formation of the supramolecular might take place between two discrete molecules. The receptor behavior of **2** was slightly affected by the presence of hydrogen sulfate anions, the NH_2_ resonances being shielded by 0.2 ppm. The ΔδNH = 0.11 ppm, the ΔδNH_2_1 = 0.13 ppm, and ΔδNH_2_2 = 0.04 ppm. It is thus expected that weak interactions are possible with this anion; the calculated binding constant is 8.94 M^−1^. For other tested anions, ClO_4_^−^, BF_4_^−^, and PF6^−^, the characteristic resonances of NH and NH_2_ protons are largely unaffected by the presence of these anions (Figures S14–S17 in the Supplementary Materials). Consequently, we deduce that the azulene-based thiosemicarbazone does not exhibit the characteristics of an effective anion receptor, a small recognition effect being observed for small-size chloride anions.

**Figure 14:**
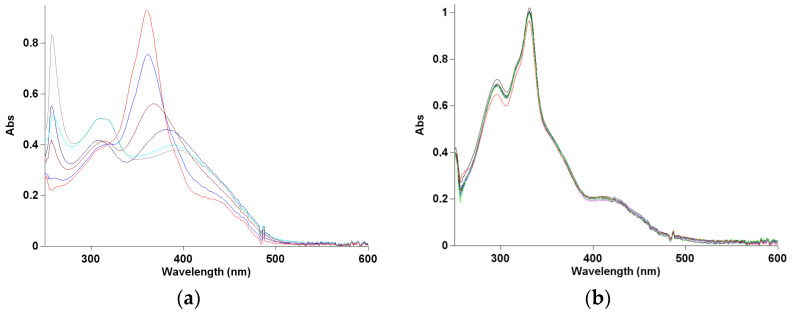
UV-Vis titration of compound **1** (1.74 × 10^−5^ M, (**a**)) and **2** (1.67 × 10^−5^ M, (**b**)) with HgCl_2_ (aqueous solution, 0, 2, 4, 6, 8, and 10 equivalents); initial spectrum—red in both cases.

However, taking into account the structural feature of the azulene-based *bis*(thiosemicarbazone), we have redirected our focus towards the recognition of metal cations. Specifically, we have selected the Hg^2+^ cation as a target, given its known toxicity and our previous findings that azulene-substituted terpyridines exhibit promising recognition properties towards this metal ion [49]. The presence of donor atoms such as nitrogen and sulfur (in thiosemicarbazones) or nitrogen and oxygen (in semicarbazones) enables these compounds to form stable coordination complexes with toxic mercury (Hg^2+^) metal ions [50]. Upon addition of a HgCl_2_ aqueous solution to a DMSO solution of **1**, the absorption maxima at 360 nm does not change in its position, with only a slight variation of the intensity (Figure 14a). Also, the ^1^H-NMR titration of **1** with HgCl_2_ (Figure S18, Supplementary Materials) shows no significant changes in the characteristic N*H* and N*H*_2_ resonances, showing that no coordination takes place. In the case of the bis-thiosemicarbazone receptor, **2**, the titration with HgCl_2_ causes significant changes in the absorption spectrum. As HgCl_2_ is gradually added, the maximum absorption band shifts bathochromicaly at 421 nm, indicative of a change in the electronic environment of the thiosemicarbazone, likely due to coordination with Hg^2+^ ions. Concurrently, a new absorption maximum appears at 320 nm, increasing in intensity as the concentration of Hg^2+^ ions increases (Figure 14b). An isosbestic point at ~300 nm at up to three equivalents of HgCl_2_ is observed, indicative of an equilibrium between the species. The calculated binding constant is 97.54 M^−1^, showing a good binding affinity for mercury(II) ions.

**Figure 15:**
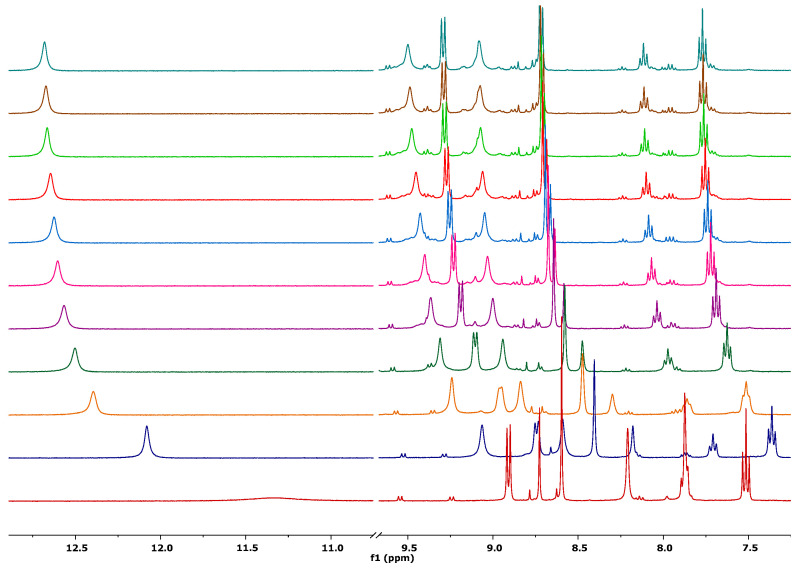
^1^H-NMR titration of **2** (10 mM) in DMSO-*d*_6_ with solid HgCl_2_ (0, 1, 2, 3, 4, 5, 6, 7, 8, 9, and 10 equivalents).

In addition to the measured electronic absorption spectra, the metallation titration of **2** with HgCl_2_ was analyzed by ^1^H-NMR spectroscopy (Figure 15). The addition of 1 equivalent of the mercury salt to a solution of **2** in DMSO-*d*_6_ (35 mM) caused a visible color change in the solution in the NMR tube. The most affected protons are the N*H_2_* (Δ*δ*NH_2_1 = 1.29 and Δ*δ*NH_2_2 = 1.21 ppm) and the azomethine protons, C*H*=N (Δ*δ* = 0.12), showing that the coordination takes place inside the cavity formed by bis(thiosemicarbazone). The N*H* protons are also affected by mercury coordination, being shifted downfield by around 1.34 ppm. Significant downfield shifts were observed also for all the azulene protons. Overall, the binding constant for the Hg^2+^ is 109.17 M^−^, similar to the UV-Vis titration experiments, showing a good affinity of **2** for this metal ion.

### 2.6. Cytotoxicity

**Figure 16:**
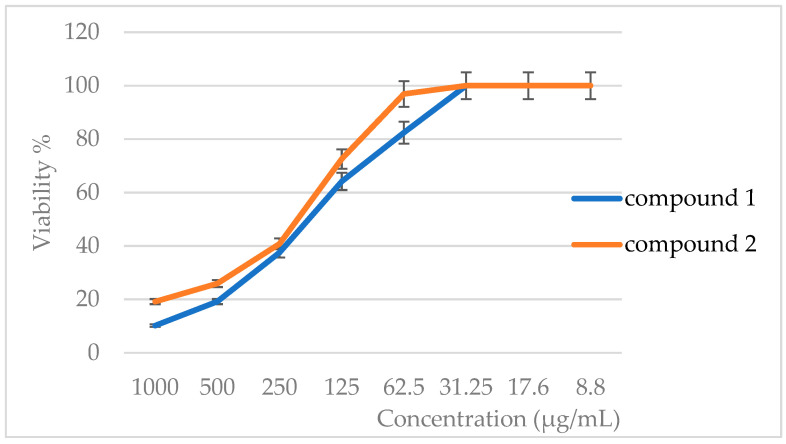
The effect of azulene-bis(semicarbazone), **1,** and azulene-bis(thiosemicarbazone), **2**, on the viability of MG63 cells.

Azulene derivatives have been investigated for their potential therapeutic applications, including anti-inflammatory effects, anticancer activities, and photoprotective properties. Moreover, bis-hydrazones from phenyl-dialdehyde demonstrated good cytotoxic action against various cell lines [51]. Thus, to assess the cytotoxicity of the compounds described herein, we evaluated their effects on osteosarcoma MG63 cells (American Type Culture Collection (ATCC) CRL-1427). Cells were exposed to azulene-based bis-(thio)semicarbazones, **1** and **2**, at concentrations ranging from 1 mg/mL to 7.8 µg/mL. Cytotoxicity was assessed using the CellTiter kit (Promega, Madison, WI, USA) and confluency assays in real-time using the Incucyte Live-Cell Analysis System. The results, presented in Figure 16, are expressed as viability percentages relative to control cells.

**Figure 17:**
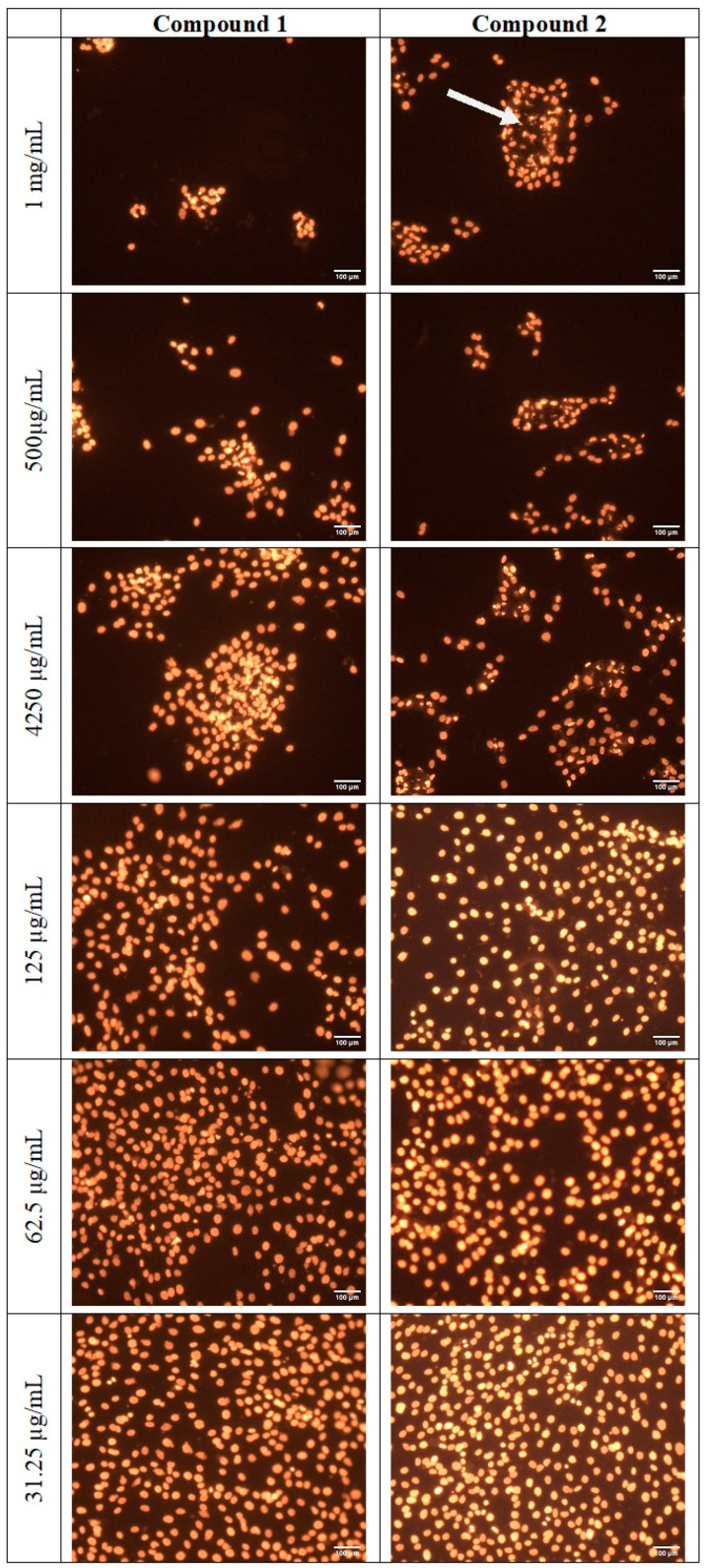
Propidium iodide staining of MG63 cells treated with different concentrations of azulene-based bis-(thio)semicarbazone; the white arrow indicates broken cells, nuclear debris that occur in the secondary apoptosis and necrosis.

The IC_50_ values of the azulene-based bis-(thio)semicarbazones were determined to be 473.08 μM (141.12 µg/mL) for compound **1** and 472.40 μM (156.10 µg/mL) for compound **2**. These compounds did not exhibit significant antiproliferative activity against osteosarcoma cell lines by comparison to the standard anticancer reference drug methotrexate (MTX), which exhibits an IC_50_ value of 26.52 μM, or phenylene (bis-hydrazone), for which the IC_50_ value was reported to be 18.20 μM [51]. However, despite their limited efficacy, the propidium iodide staining revealed a concentration-dependent decrease in cell number, accompanied by characteristic morphological changes indicative of apoptosis and necrosis (Figure 17).

## 3. Experimental

### 3.1. Materials and Methods

All reagents were used as received. The solvents were spectroscopic grade. First, azulene-1,3-dicarboxaldehydes was obtained following the Vilsmeier reaction [52]. The high-resolution mass spectra were acquired with a QTOF LCMS-9030 mass spectrometer from Shimadzu (Kyoto, Japan), equipped with the ESI interface and auxiliary interface for calibration. The calibration was performed with a sodium iodide solution. Samples dissolved in acetonitrile at a concentration of 1 ppm were infused directly into the ESI interface with an LC-40D ×3 pump at a flow rate of 0.4 mL/min with a mobile water phase (0.1% formic acid) 10% and the acetonitrile. The sample solutions were loaded into a loop using a SIL-40C ×3 automatic injector. The injected volume was 1 μL. At the ESI interface, the nebulizing gas was nitrogen with a flow rate of 3 L/min, nitrogen was used as the drying gas with a flow rate of 10 L/min, the heating gas was air at a rate of 15 L/min, and the temperature of the ESI interface was 250 °C. Melting points were determined using a Krüss Optronic instrument, Hamburg, Germany, model KSPI. The NMR spectra were recorded on a Bruker Avance II 500 MHz (^1^H: 500 MHz, ^13^C: 175.47 MHz), Karlsruhe, Germany. The structural assignment of the ^1^H and ^13^C resonances was obtained from ^1^H COSY and ^13^C HSQC, and HMBQ experiments. NMR titrations with HgCl_2_ were performed for a 0.6 mL solution of the receptor (around 10 mM) in DMSO-*d*_6_ with solid mercury salt from 1 to 10 equivalents. The same procedure was followed for the titrations with tetrabutylammoniun anions. The association constants were calculated using the http://app.supramolecular.org/bindfit/ software [48]. The electrochemical experiments were conducted in 1 mM solutions in DMSO, with 0.1 M Bu_4_NClO_4_ serving as the electrolyte. A three-electrode setup was employed, consisting of a platinum working electrode, a silver/silver chloride reference electrode, and a graphite counter electrode. The measurements were performed using an Autolab PGSTAT30 potentiostat from Metrohm, Herisau, Switzerland. The IR spectra were obtained using a Bruker 70 Tensor instrument, Borken, Germany in the KBr pellets. The fluorescence quantum yield was determined by comparative measurement with a dilute solution of quinine bisulfate in 0.1 N sulfuric acid (H_2_SO_4_), which has a known absolute quantum yield of 0.55 [53]. The fluorescence spectra were recorded with a Jasco FP-6500 spectrofluorometer (Jasco Europe, Cremella, Italy) equipped with a 150 W Xenon lamp. The excitation wavelength was 350 nm for working concentrations of 3.48 × 10^−6^ M (compound **1**) and 3.34 × 10^−6^ M (compound **2**). UV–vis spectra were recorded on a Varian Cary 100 spectrophotometer using 1 cm quartz cells. The UV-Vis titrations were performed as constant host titrations (1.74 × 10^−5^ M for compound **1** and 1.67 × 10^−5^ M for compound **2**) in DMSO with HgCl_2_ (4.95 mM aqueous solution, 0, 2, 4, 6, 8, and 10 equivalents DMSO) and the respective TBAX stock solution of ~5 mM in DMSO at room temperature.

X-ray diffraction measurements were performed on an STOE IPDS II diffractometer (STOE & Cie GmbH, Darmstadt, Germany), operating with a Mo Kα (λ = 0.71073 Å) X-ray tube with a graphite monochromator. The structures were solved by direct methods and refined by full-matrix least-squares techniques based on F^2^ [54]. The non-H atoms were refined with anisotropic displacement parameters. Atomic scattering factors were taken from the international tables for X-ray crystallography. Hydrogen atoms were included but not refined. Calculations were performed using the SHELX-2014 crystallographic software package (version 1). Drawings of the molecules were performed with the program Diamond 4. A summary of the crystallographic data and the structure refinement are given in Table 1. Crystallographic data (excluding structure factors) have been deposited with the Cambridge Crystallographic Data Centre with CCDC reference number 2400616. These data can be obtained free of charge via http://www.ccdc.cam.ac.uk/structures (accessed on 20 December 2024), or from the Cambridge Crystallo-graphic Data Centre, 12 Union Road, Cambridge, CB2 1EZ, UK; fax: (+44)-1223-336-033; or e-mail: deposit@ccdc.cam.ac.uk.

### 3.2. General Procedure for the Synthesis of Compounds ***1*** and ***2***

Azulene-1,3-dicarboxaldehyde (184 mg, 1 mmol) was added to a solution of semicarbazide/thiosemicarbazide (2.5 equivalents, 2.5 mmol) in ethanol (50 mL), followed by addition of 5 drops of acetic acid glacial. The resulting solution was refluxed overnight with continuous stirring, monitoring the disappearance of the dialdehyde precursor by thin-layer chromatography. After reaction completion, the mixture was filtered and washed with ethanol and diethyl ether to yield pure products. IR, ^1^H NMR, and ^13^C NMR were used to determine the structure of the compounds.

*(2E,2′E)-2,2′-(azulene-1,3-diylbis(methaneylylidene))bis(hydrazine-1-carboxamide)*, **1**, was synthesized following the general procedure using semicarbazide (188 mg, 2.5 mmol). After removal of the solvent, the obtained dark green solid was washed with ethanol and diethyl ether. There were isolated 230 mg (0.77 mmol) green powder, 77% yield; m.p > 300 °C with decomposition; a transition phase was observed at 223–225 °C; ^1^H NMR: (DMSO-*d*_6_, 500 MHz): δ 10.17 (s, 2H, NH), 8.81 (d, 2H, *J* = 9.8 Hz, H-8 and H-4), 8.61 (s, 1H, H-2), 8.38 (s, 2H, CH=N), 7.77 (t, 1H, *J* = 9.8 Hz, H-6), 7.37 (t, 1H, *J* = 9.8 Hz, H-5 and H-7), and 6.49 (bs, 4H, NH_2_) ppm. ^13^C-NMR (DMSO-*d*_6_, 125 MHz): δ 156.89 (*C*=O), 140.36 (*C*H-6), 138.36 (*C*q-9/10), 136.10 (*C*H-4/8), 135.95 (*C*H-2), 135.55 (*C*H=N), 126.54 (*C*H-5/7), 123.46 (*C*q-1/3) ppm. UV-Vis (DMSO):296 (lgɛ = 4.63), 330 (lg ɛ = 4.79), 410 (lg ɛ = 4.10). Selected IR: 3470, 2884, 2827, 1676, 1577, 1433, 1400, 1111, 1045, 746 cm^−1^. MS: 299.12532 [M + H]^+^ (calcd. for C_14_H_14_N_6_O_2_ 299.12510).
*(2E,2′E)-2,2′-(azulene-1,3-diylbis(methaneylylidene))bis(hydrazine-1-carbothioamide)*, **2**, was synthesized following the general procedure using thiosemicarbazone (230 mg, 2.5 mmol). After removal of the solvent, the green solid was washed with ethanol (3 × 10 mL) and diethyl ether for drying. There were isolated 238 mg (72 mmol) light green powder, 72% yield; m.p = 268–269 °C; ^1^H NMR: (DMSO-*d*_6_, 500 MHz): δ 11.36 (s, 2H, NH), 8.91 (d, 2H, *J* = 9.8 Hz, H-8 and H-4), 8.72 (s, 1H, H-2), 8.60 (s, 2H, CH=N), 8.19 (bs, 2H, NH_2_), 7.89–7.85 (m, 3H, NH_2_ and H-6), and 7.51 (t, 1H, *J* = 9.8 Hz, H-5 and H-7) ppm. ^13^C-NMR (DMSO-*d*_6_, 125 MHz): δ 177.13 (*C*=S), 140.83 (*C*H-6), 139.64 (*C*q-9/10), 138.52 (*C*H=N), 137.72 (*C*H-2), 136.70 (*C*H-4/8), 127.87 (*C*H-5/7), 122.90 (*C*q-1/3) ppm. UV-Vis (DMSO):360 (lg ɛ = 4.90), 410 (lg ɛ = 4.22). Selected IR: 3440, 3262, 3151, 3033, 2991, 2885, 2814, 1591, 1537, 1388, 831 cm^−1^. MS: 331.07944 [M + H]^+^ (calcd. for C_14_H_14_N_6_S_2_ 331.07941).

### 3.3. Determination of Cell Viability by the CellTiter Assay

Cell viability was determined using the CellTiter 96^®^ AQueous One Solution Cell Proliferation Assay kit (Promega, Madison, WI, USA) according to the manufacturer’s instructions. Thus, cells were seeded in a 96-well plate at a density of 7500 cells per well and incubated at 37 °C, 5% CO_2_ to allow for adherence and proliferation. After 24 h, the cells were treated with binary dilutions of azulene derivates starting from mg/mL. After incubation, 10 µL of cell titer solution was added to each well, and the plate was further incubated for 24 h. The absorbance of the solution was measured at 490 nm using a spectrophotometer (e.g., a plate reader). The test is based on reducing a tetrazole compound (MTS) in the mitochondria of viable cells. It leads to the formation of a colored formazan product, soluble in the medium, the amount of which is directly proportional to the number of viable cells. Data were normalized to the sample, to which no MTS was added, and expressed as a percentage of the untreated control. Wells without MTS were subsequently fixed with ethanol 70, stained with propidium iodide (PI), and photographed under a Zeiss Observer D1 microscope, Oberkochen, Germany equipped with fluorescence mode.

### 3.4. Determination of Cell Viability Using the Incucyte System

The Incucyte^®^ Live-Cell Analysis System (Sartorius, Göttingen, Germany) assessed cell viability in real-time. Cells were seeded in a 96-well plate at 7500 cells per well density and incubated at 37 °C and 5% CO_2_ for 24 h. The cells were treated with binary dilutions of compounds ranging from 1 mg/mL to 7.8 µ/mL. The Incucyte system continuously monitored the cells for 72 h, capturing images at regular 12 h intervals. Cell viability was determined using the cell confluency assay (the degree of coverage of the well surface by cells) as an indirect measure of cell growth and proliferation. The obtained images were automatically analyzed using Incucyte software S3 (Sartorius, Goettingen, Germany), which generated growth curves as a function of incubation time. Cell viability was expressed relative to the untreated control group. Data were exported and processed for further statistical analysis.

## 4. Conclusions

In summary, we have synthesized two azulene-based hydrazones which were obtained by Schiff-based condensation reaction of azulene-1,3-dicarboxaldehyde with semi- and thiosemicarbazide in the presence of acetic acid. These compounds showed fluorescence properties with emission centered at 470 nm upon excitation at a 350 nm wavelength. Both compounds are redox active with reduction peaks observed at approximately −0.8 V, attributed to the reduction of the imine double bond, and at −0.60 V, ascribed to the reduction of the azulene moiety. Taking advantage of the visible absorption band of azulene derivatives, we tested the two for their ability to recognize anions and poisoning metal cations, in particular, mercury (II) cations. The azulen-1,3-bis(semicarbazone) showed no real recognition properties; instead, the azulen-1,3-bis(thiosemicarbazone) compound exhibited a weak recognition effect for chloride anions and a good binding affinity for mercury(II) ions. The azulene-containing bis-hydrazone was tested against osteosarcoma MG63 cells with no significant antiproliferative activity. However, a concentration-dependent decrease in cell number was observed by propidium iodide staining.

## Figures and Tables

**Table 1:** Crystallographic data for compound **2**.

Chemical formula	C_18_H_29_N_3_OS
Molar mass (g mol^−1^)	335.50
*T* (K)	100
Crystal system	monoclinic
Space group	*P*2_1_/*c*
*a* (Å)	18.4754(14)
*b* (Å)	9.2040(7)
*c* (Å)	11.5714(9)
*α* (°)	90
*β* (°)	95.517(2)
*γ* (°)	90
*V* (Å^3^)	1958.6(3)
*Z*	4
*ρ*calc (g cm^−3^)	1.138
*μ* (mm^−1^)	0.173
*F*(000)	728.0
Crystal size (mm)	0.500 × 0.200 × 0.150
Radiation (λ/Å)	Mo-K*α* (*λ* = 0.71073)
2*θ* range (°)	4.43–57.282
Reflections collected	37828
*R*int	0.0661
GOF on *F*2	1.051
*R*1, *wR*2 (*I* ≥ 2*σ* (*I*))	0.0403, 0.0909

**Table 2:** Selected bond lengths and hydrogen bond metrics for compound **2**.

Selected bond lengths (Å) for compound **2**						
C1-S1 = 1.691(3)		C1-N2 = 1.341(4)			C14-S2 = 1.688(3)	
C1-N1 = 1.339(4)		C2-N3 = 1.284(4)			C14-N4 = 1.320(4)	
N3-N2 = 1.391(3)		N6-N5 = 1.386(3)			C14-N5 = 1.342(4)	
					C13-N6 = 1.284(4)	
Hydrogen bond metrics for compound **2**						
D-H···A	d(DH)/Å		H···A/Å	D···A/Å		(D-H···A)/°
N4-H3N···S1a	0.82		2.76	3.470		144.6
N1-H1N···S1a	0.92		2.65	3.433		142.1
N5-H5A···S2c	0.86		2.44	3.287		168.5
N2-H2A···O1d	0.86		2.14	2.902		148.6
N4-H4N···O2b	0.96		2.19	2.879		178.9

## Data Availability

Data are contained within the article and Supplementary Materials.

## Supplementary Materials

The following supporting information can be downloaded at: https://www.mdpi.com/article/10.3390/molecules30010083/s1: spectroscopic data, titration experiments, complementary electrochemical experiments, and cif file for compound **2**.
