# Supplementary material for: Synthesis, Physicochemical Properties, and Ion Recognition Ability of Azulene-Based Bis-(Thio)Semicarbazone
Source: Molecules. 2024 Dec 29;30(1):83. doi: 10.3390/molecules30010083 (PMC11721881; doi:10.3390/molecules30010083)
Supplement: Supplementary file 1 [file molecules-30-00083-s001.zip › molecules-3326368-supplementary.pdf]

Synthesis, Physicochemical Properties, and Ion Recognition Ability of Azulene-Based Bis-(Thio)Semicarbazone

Anamaria Hanganu [1,2], Catalin Maxim [1,2], Andreea Dogaru [1], Adrian E. Ion [1], Coralia Bleotu [3,4], Augustin M. Madalan [2], Daniela Bala[2] and Simona Nica [1,*]

1 "C. D. Nenitzescu" Institute of Organic and Supramolecular Chemistry, Splaiul Independentei 202B, 060023 Bucharest, Romania; anamaria_hanganu@yahoo.com (A.H.); catalin.maxim@chimie.unibuc.ro (C.M.); g.a.dogaru@gmail.com (A.D.); eugeniu_ion@yahoo.com (A.E.I.)

2 Faculty of Chemistry, University of Bucharest, 4-12 Bvd. Regina Elisabeta, 030018 Bucharest, Romania; catalin.maxim@chimie.unibuc.ro (C.M.); augustin.madalan@chimie.unibuc.ro (A.M.M.); dbala@gw-chimie.math.unibuc.ro (D.B.); anamaria_hanganu@yahoo.com (A.H.).

3 Stefan S. Nicolau Institute of Virology, 285 Mihai Bravu Avenue, 030317 Bucharest, Romania; cbleotu@yahoo.com

4 Department of Botany and Microbiology, Faculty of Biology, University of Bucharest, 1-3 Aleea Portocalelor, 060101 Bucharest, Romania

* Correspondence: simona.nica@icoscdn.ro; Tel.: +40-213-16-79-00

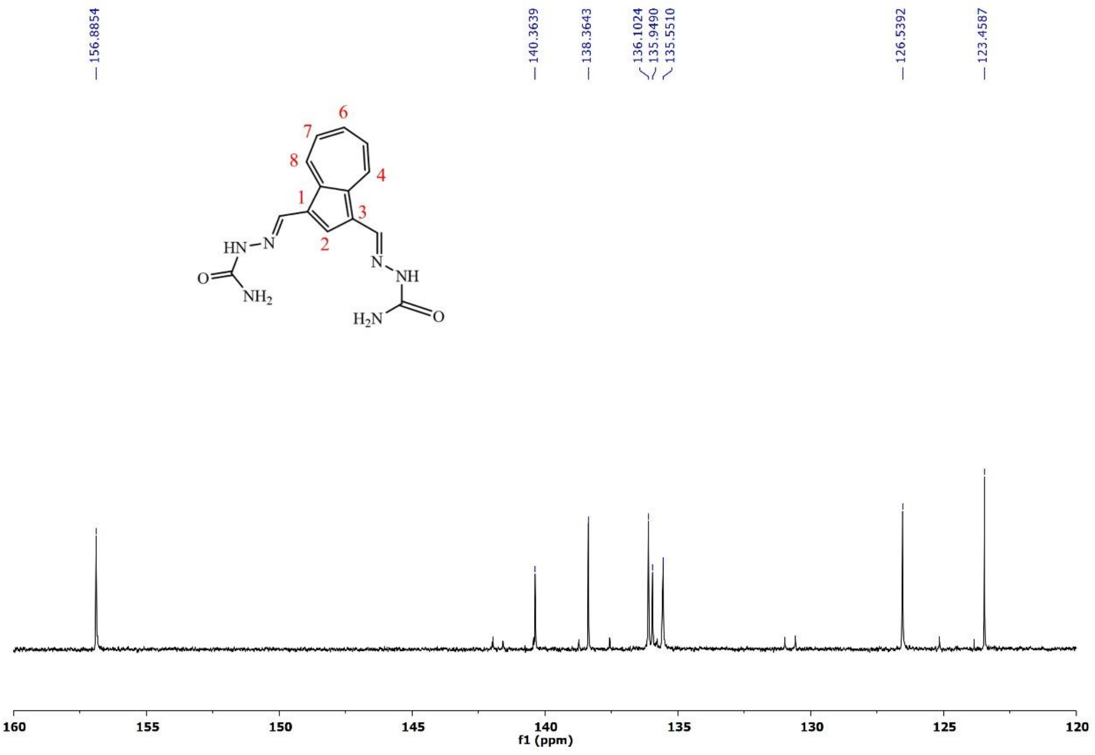

Figure S1. ^{13}C-NMR spectrum of **1** in DMSO-d$_6$ at 125 MHz

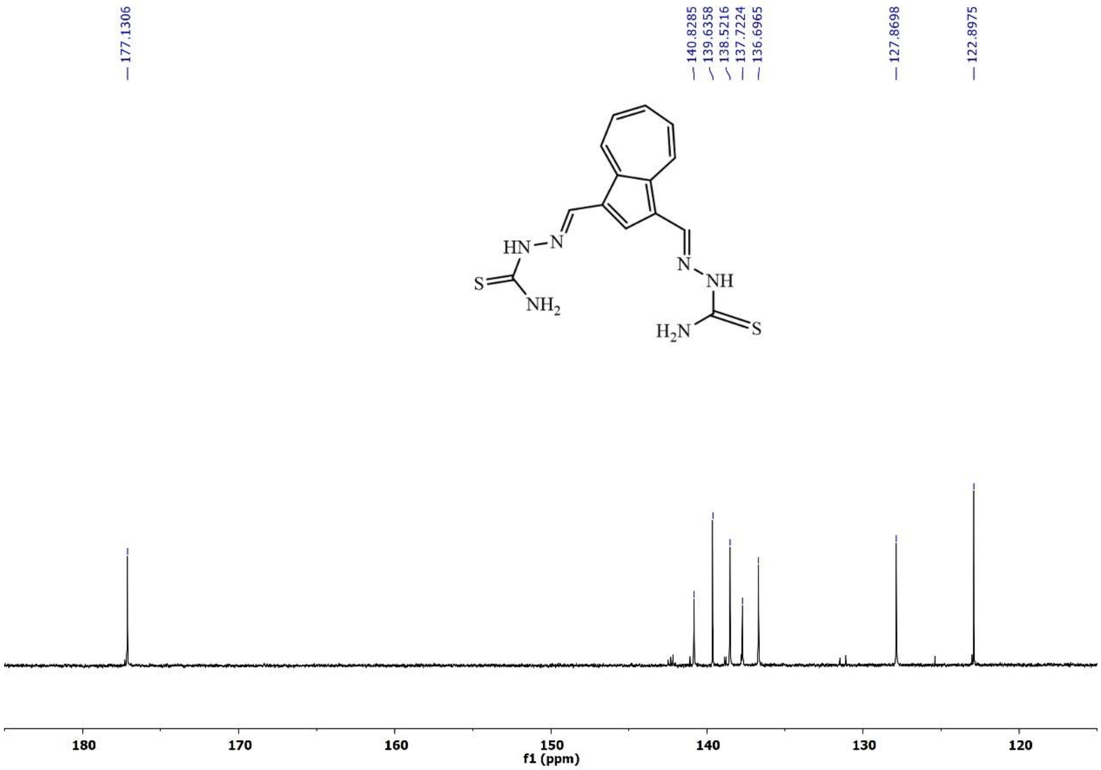

Figure S2. ^{13}C-NMR spectrum of **2** in DMSO-d_6 at 125 MHz

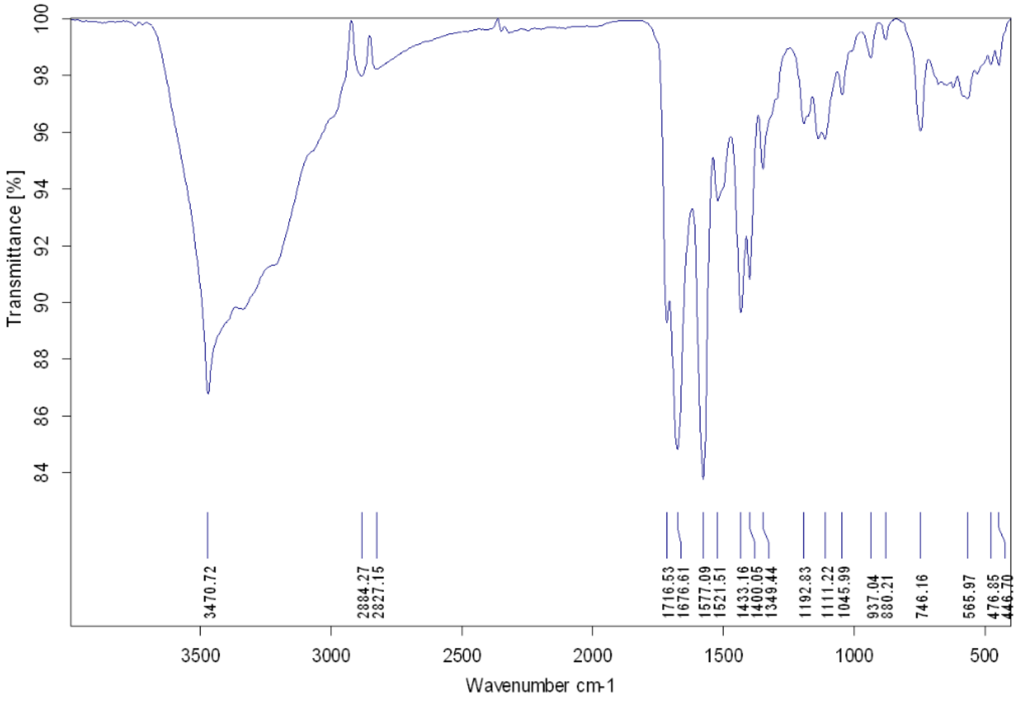

Figure S3. IR spectrum of **1**

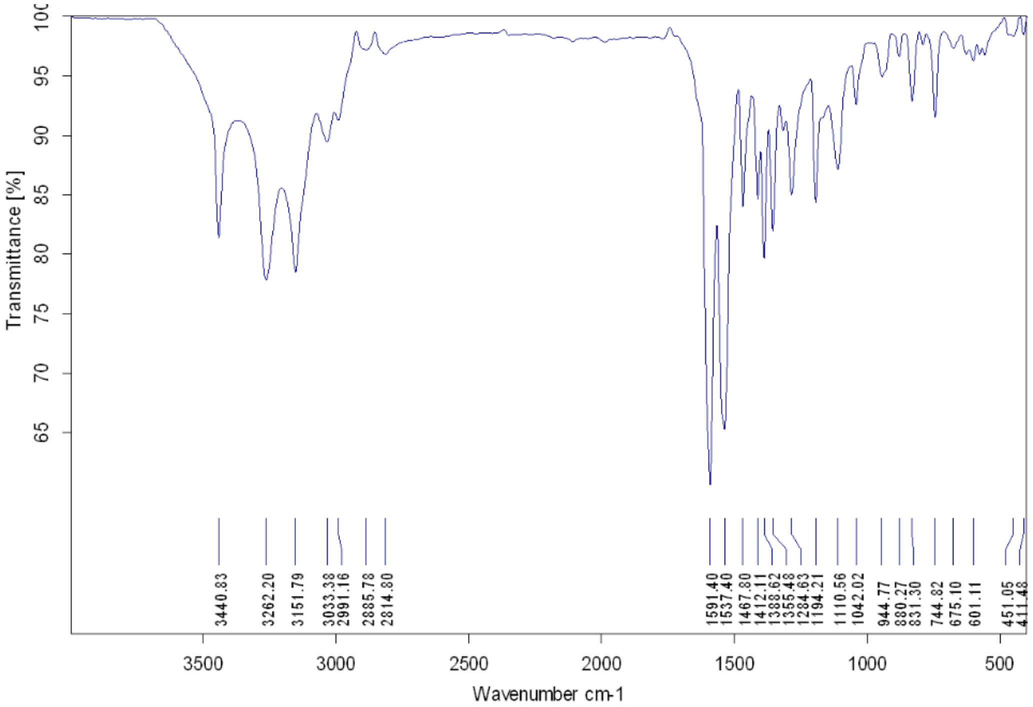

Figure S4. IR spectrum of **2**

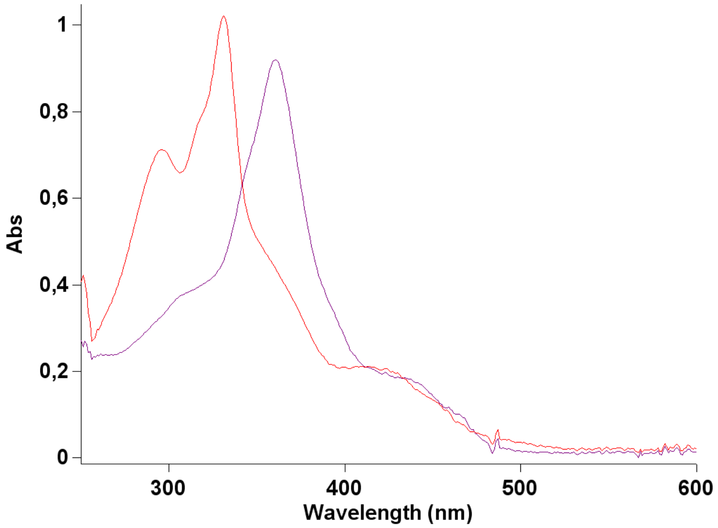

Figure S5. Electronic absorption spectra of **1** (1.74 × 10^{-5} M, red line) and **2** (1.67 × 10^{-5} M, magenta line) in DMSO, at room temperature.

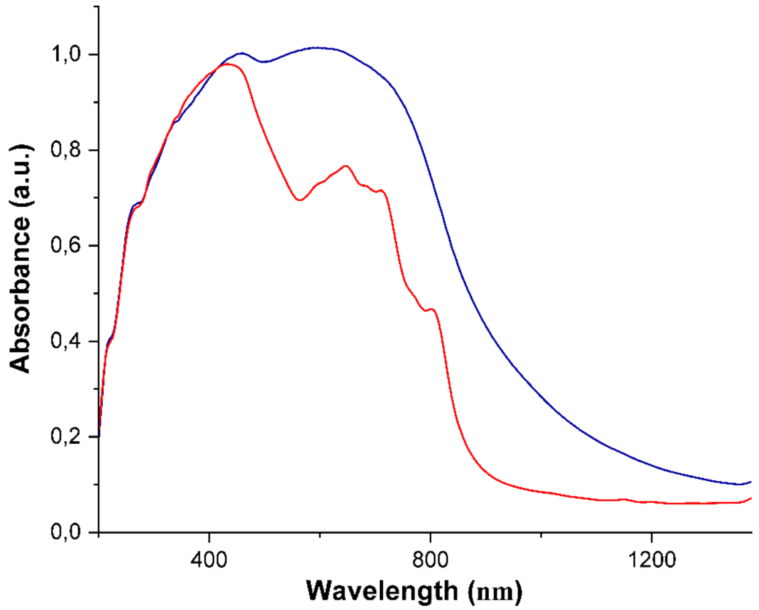

Figure S6. Solid UV-Vis spectra of compound **1** (blue) and compound **2** (red)

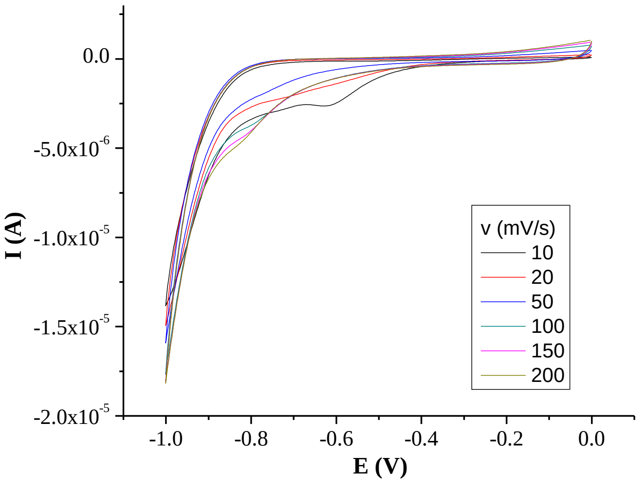

Figure S7. The electrochemical behaviour of compound **1** over the potential range [0 - (-1) -0].

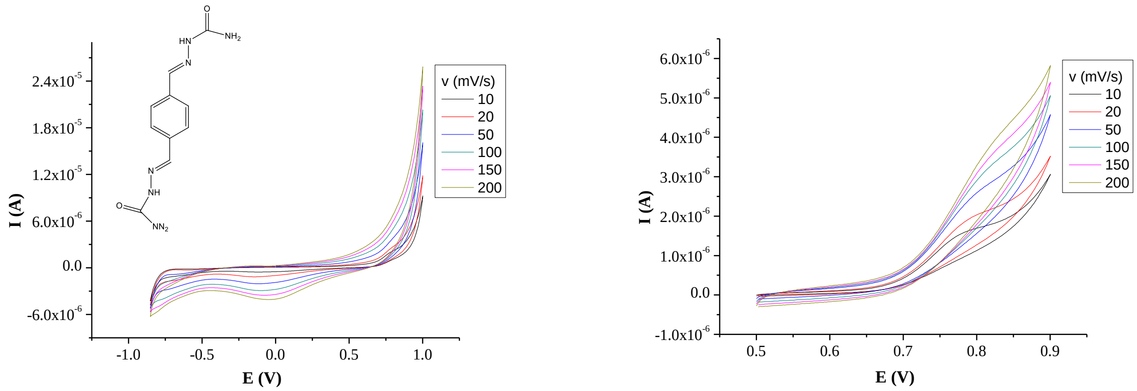

Figure S8. The electrochemical behaviour of related phenyl-1,4-bis(thiosemicarbazone) over the potential range [0 – 1– (-0.9)] V (left) and [0.5 – (0.9) – 0.5] (right).

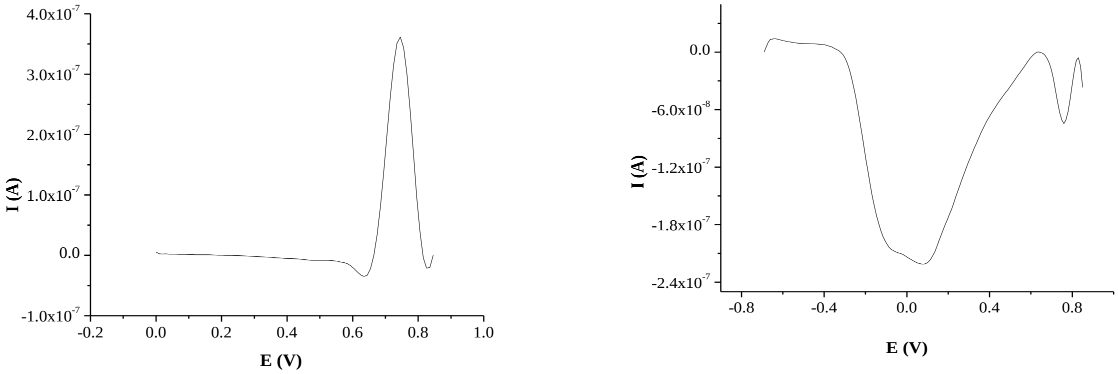

Figura S9. DPV-traces of 1 mM related phenyl-1,4-bis(thiosemicarbazone) in DMSO, with SP = 10 mV and MA = 25 mV in the 0.0 V to 0.85 V (a), and 0.85 to -0.8 V (b) potential range, scan rate 20 mV/s.

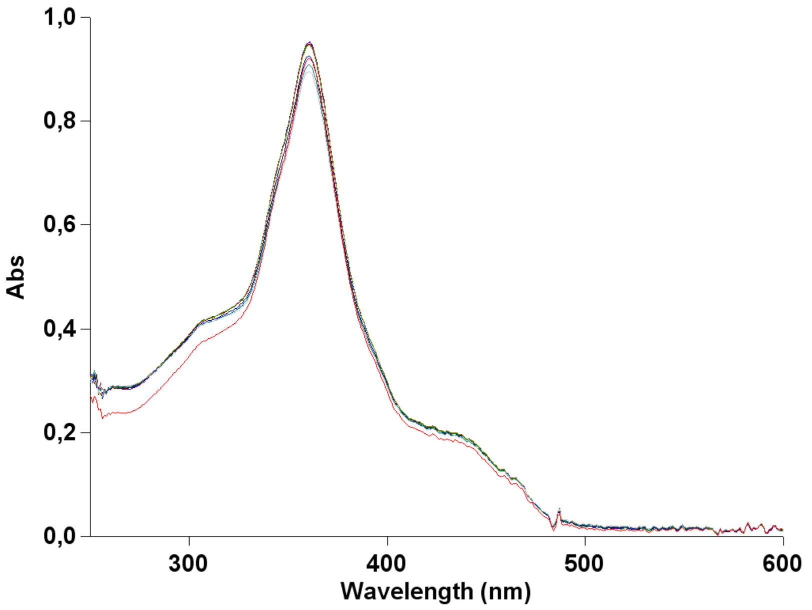

Figure S10. UV-vis titration of **2** (1.67×10^{-5} M)in DMSO with 20 equivalents of tetra-butylammonium chloride (TBACl), aliquots of ~ 5 mM stock solution (selected spectra are shown).

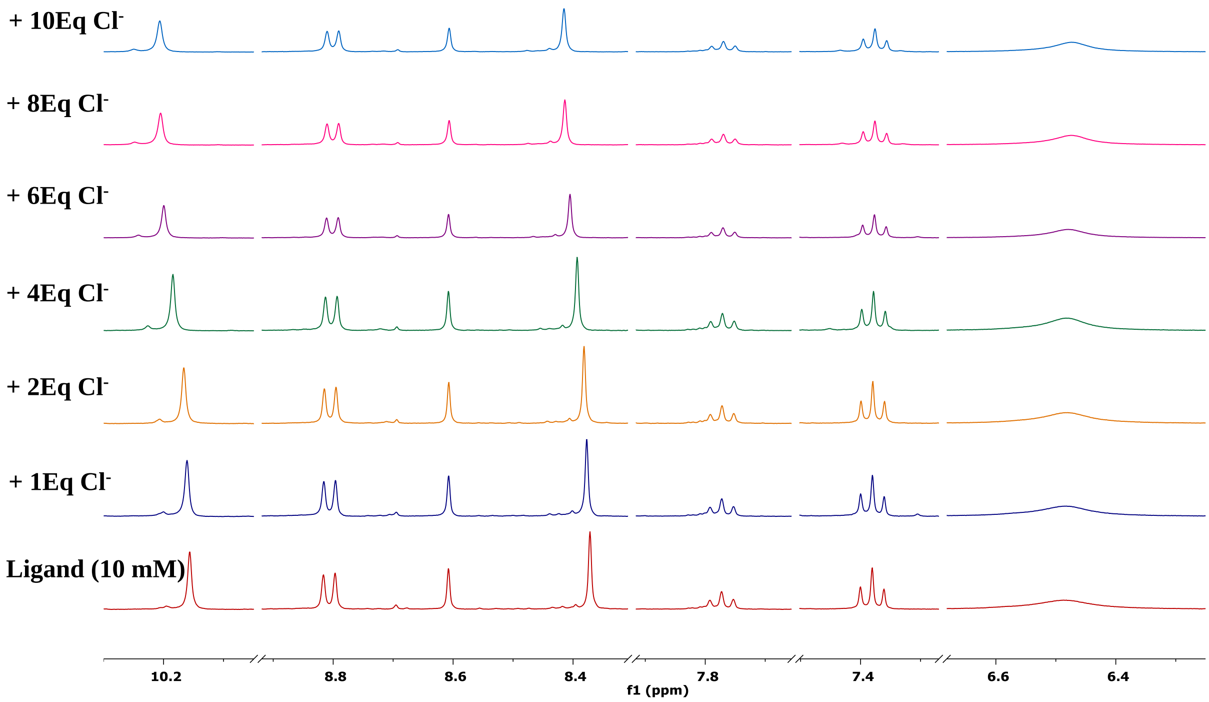

Figure S11. ^{1}H-NMR spectra of **1** (10 mM) in DMSO-d_6 of free ligand and after addition of 1, 2, 4, 6, 8 and 10 equiv of TBACl

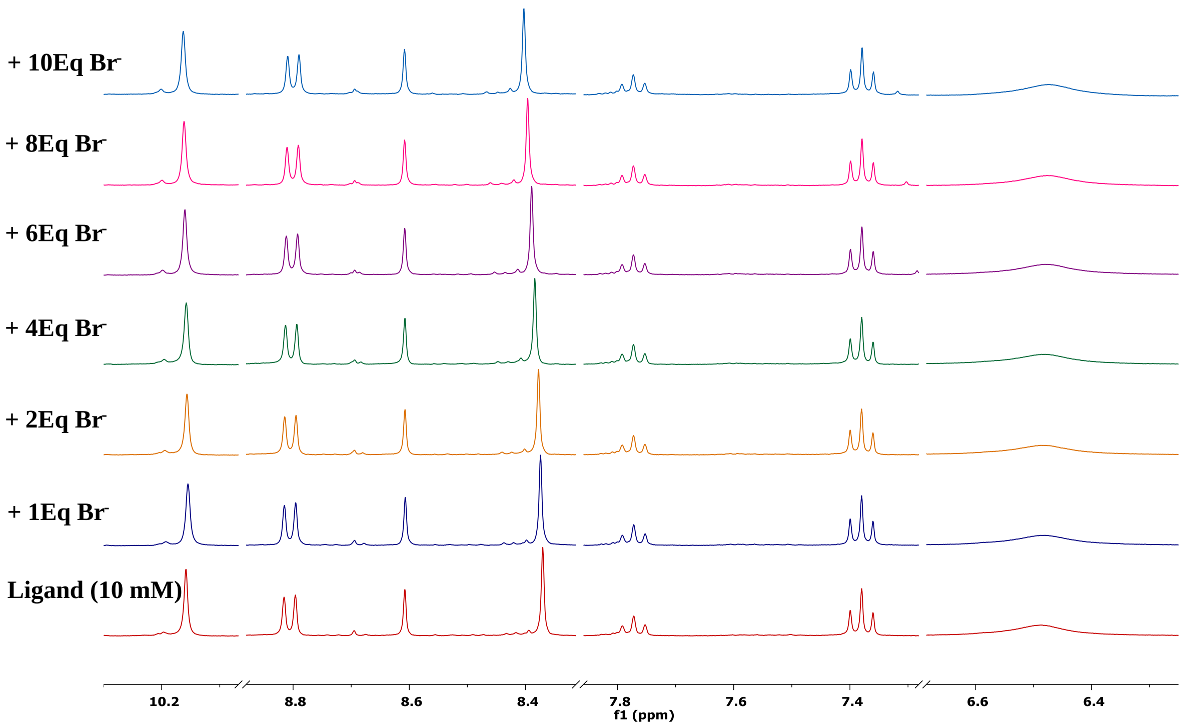

Figure S12. ^{1}H-NMR spectra of **1** (10 mM) in DMSO-d_6 of free ligand and after addition of 1, 2, 4, 6, 8 and 10 equiv of TBABr

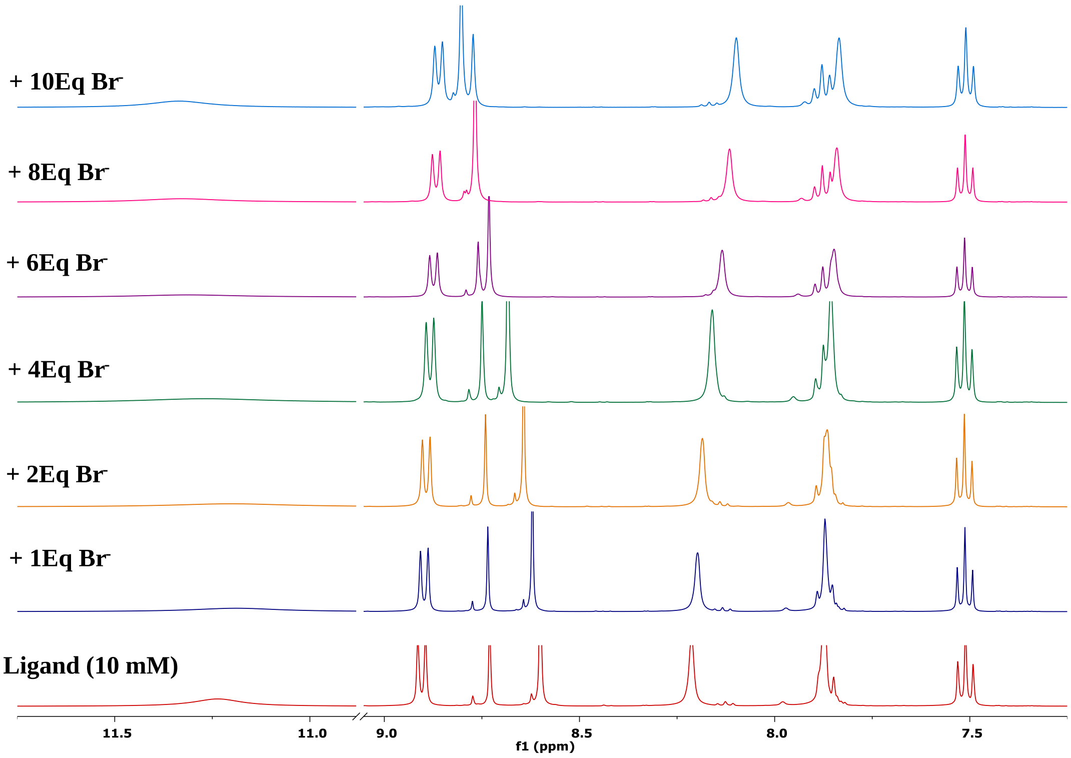

Figure S13. ^{1}H-NMR spectra of **2** (10 mM) in DMSO-d_6 of free ligand and after addition of 1, 2, 4, 6, 8 and 10 equiv of TBABr

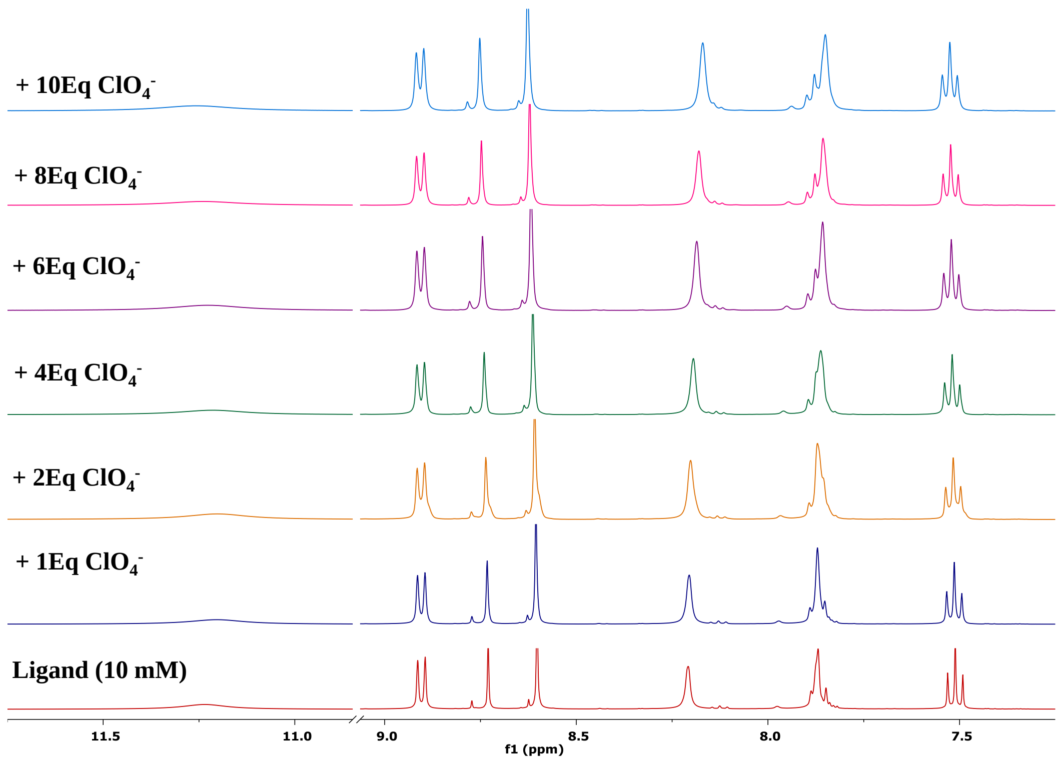

Figure S14. ^{1}H-NMR spectra of **2** (10 mM)in DMSO-d_6 of free ligand and after addition of 1, 2, 4, 6, 8 and 10 equiv of $TBAClO_4$

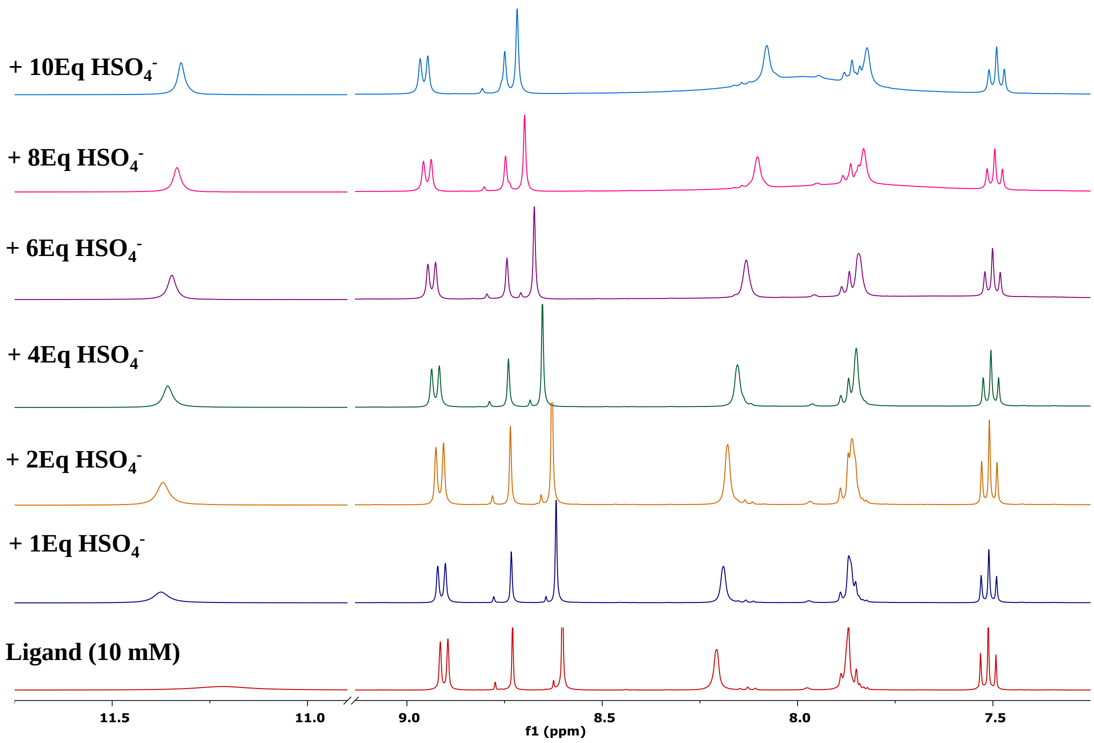

Figure S15. ^{1}H-NMR spectra of **2** (10 mM) in DMSO-d_6 of free ligand and after addition of 1, 2, 4, 6, 8 and 10 equiv of $TBAHSO_4$.

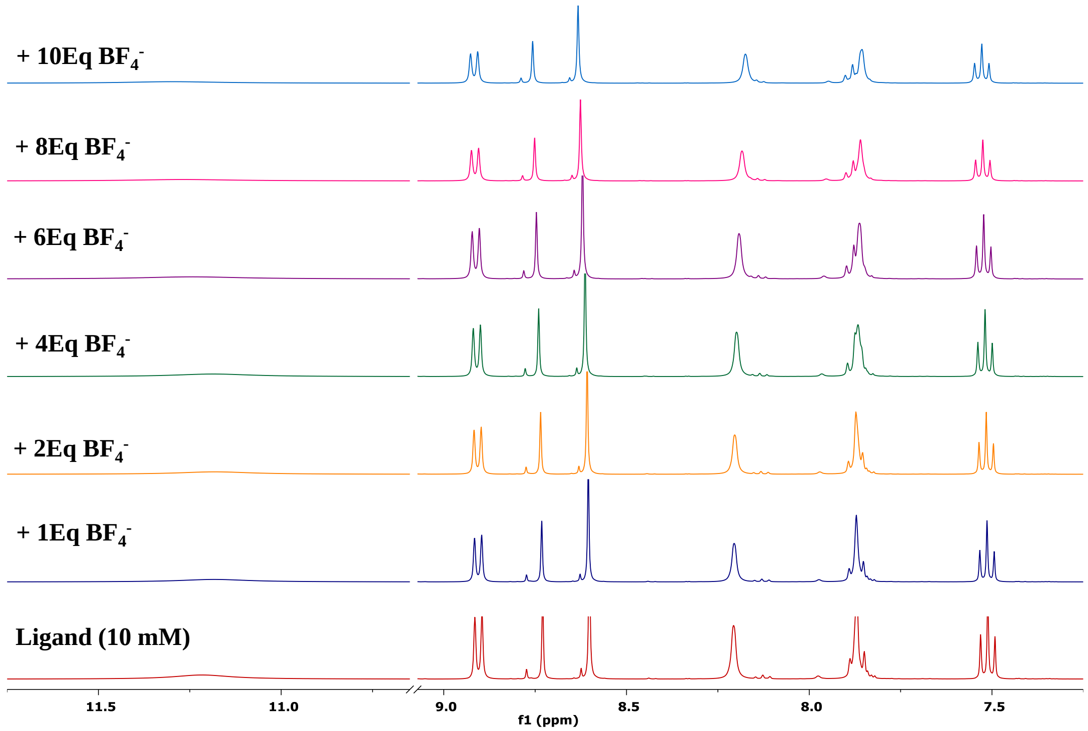

Figure S16. ^{1}H-NMR spectra of **1** (10 mM) in DMSO-d$_6$ of free ligand and after addition of 1, 2, 4, 6, 8 and 10 equiv of $TBABF_4$

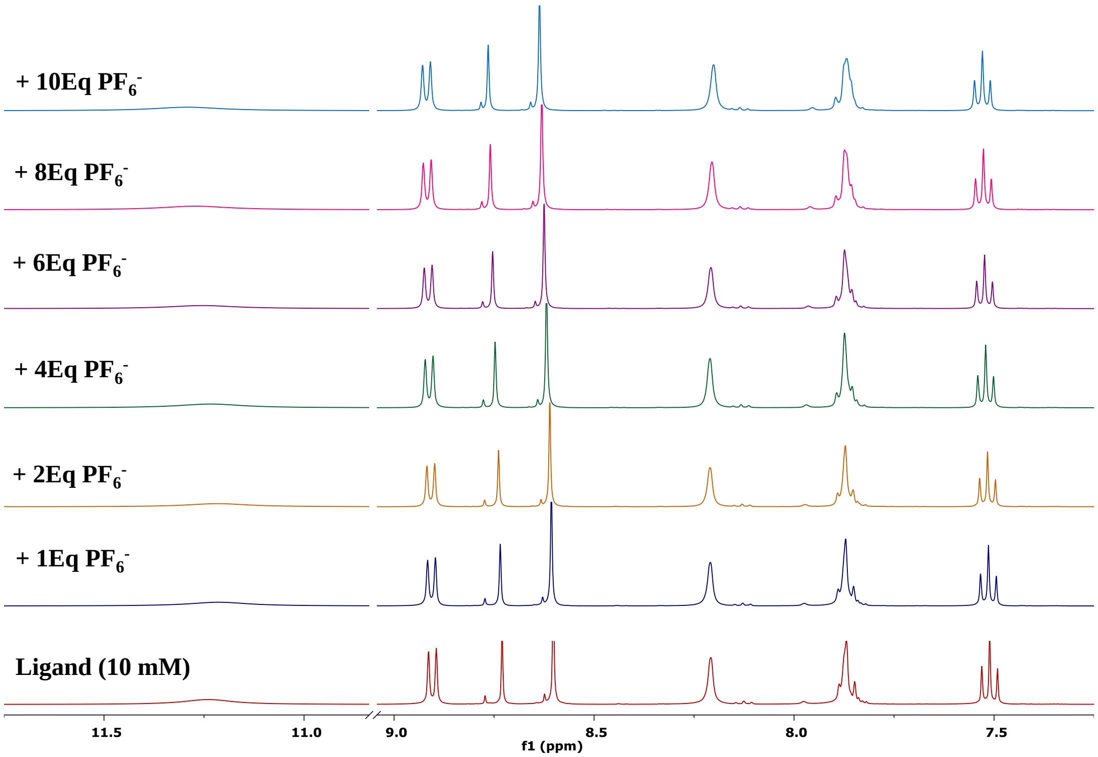

Figure S17. ^{1}H-NMR spectra of **2** (10 mM) in DMSO-d$_6$ of free ligand and after addition of 1, 2, 4, 6, 8 and 10 equiv of $TBAPF_6$

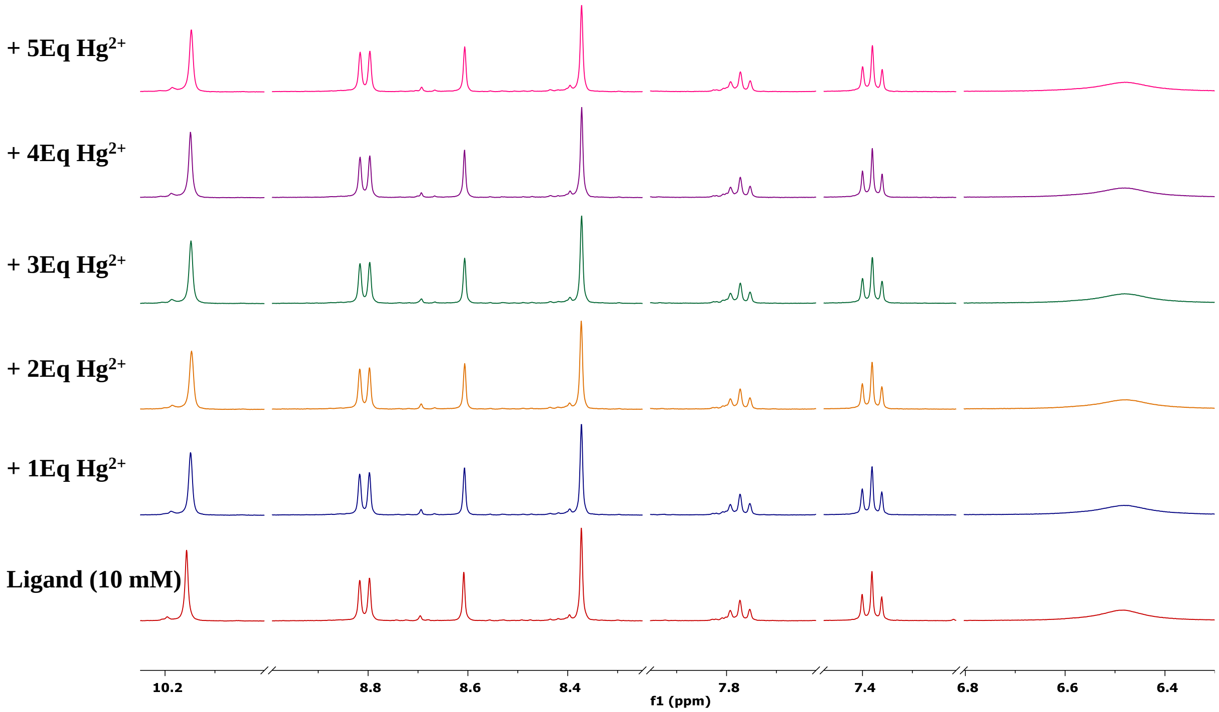

Figure S18. ^{1}H-NMR spectra of **1** (10 mM) with $HgCl_2$ (0, 1, 2, 4, 6, 8 and 10 equivalents) in DMSO-d_6 at room temperature (500 MHz).

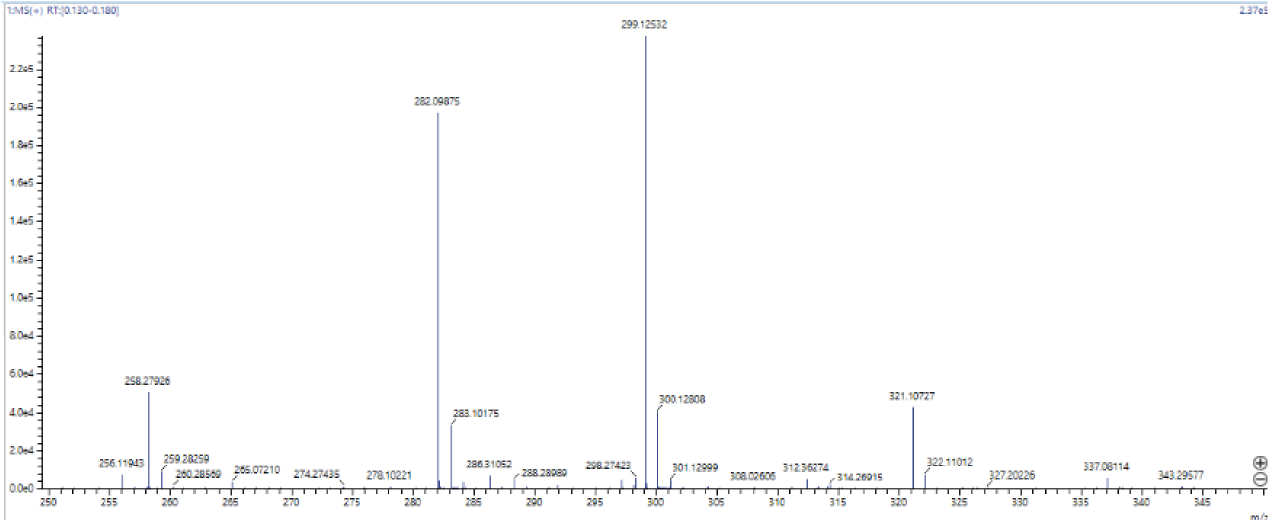

Figure S19. The high-resolution mass spectra of compound **1**.

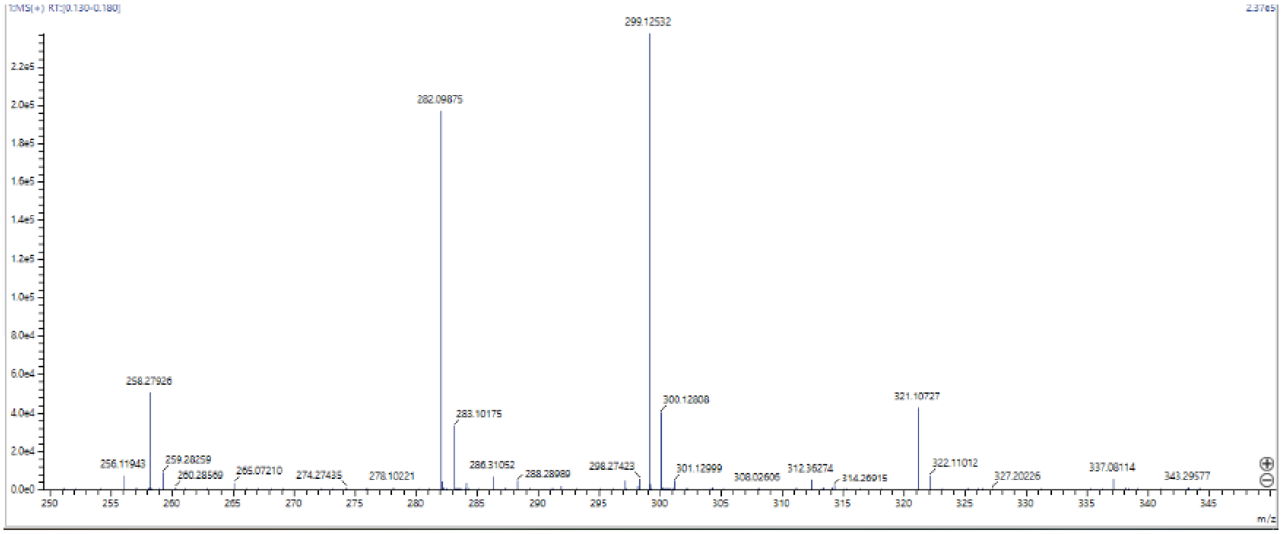

Figure S20. The high-resolution mass spectra of compound **2**.
